# Supplementary material for: Nascent RNA sequencing identifies a widespread sigma70-dependent pausing regulated by Gre factors in bacteria
Source: Nat Commun. 2021 Feb 10;12:906. doi: 10.1038/s41467-021-21150-2 (PMC7876045; doi:10.1038/s41467-021-21150-2)
Supplement: Supplementary file 1 — Supplementary information [file 41467_2021_21150_MOESM1_ESM.docx]

**Supplementary Information**

**Title**

**Nascent RNA sequencing identifies a widespread sigma70-dependent pausing regulated by Gre factors in bacteria**

Zhe Sun^1^, Alexander V. Yakhnin^1^, Peter C. FitzGerald^2^, Carl E. Mclntosh^2^, Mikhail Kashlev^1*^

^1^RNA Biology Laboratory, National Cancer Institute, National Institutes of Health, Frederick, MD 21702, USA.

^2^Genome Analysis Unit, National Cancer Institute, National Institutes of Health, Bethesda, MD 20892, USA.

^*^Correspondence:

Tel: +1-301-846-1798

Fax: +1-301-846-6988

Email: [kashlevm@mail.nih.gov](mailto:kashlevm@mail.nih.gov)


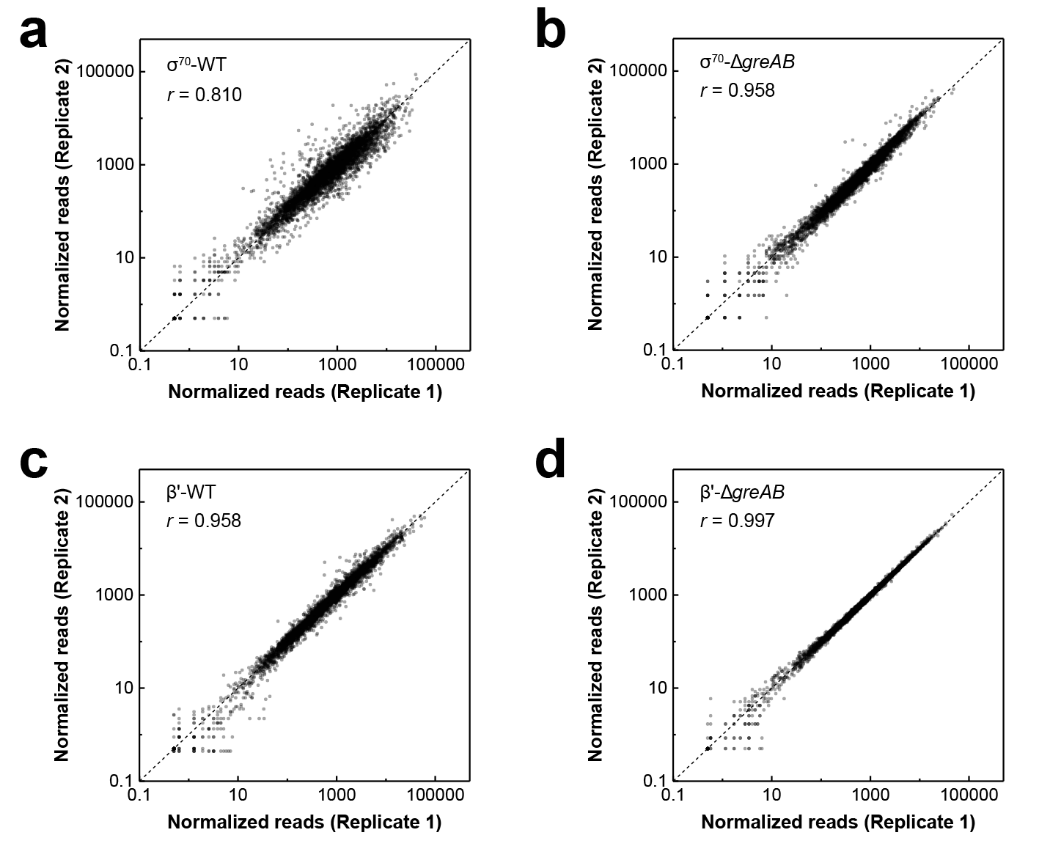


**Supplementary Fig. 1 Scatterplots showing a correlation between two biological replicates of RNET-seq from the different *E. coli* strains.** The total count of uniquely mapped reads for each *E. coli* gene from two biological replicates was normalized to the same library size (10^7^ reads). 0.5 count was manually added to the poorly expressed genes containing no mapped reads to obtain all data points and avoid bias in a log scale. The corresponding strain name is labeled in each figure. *r*, Pearson correlation coefficient.


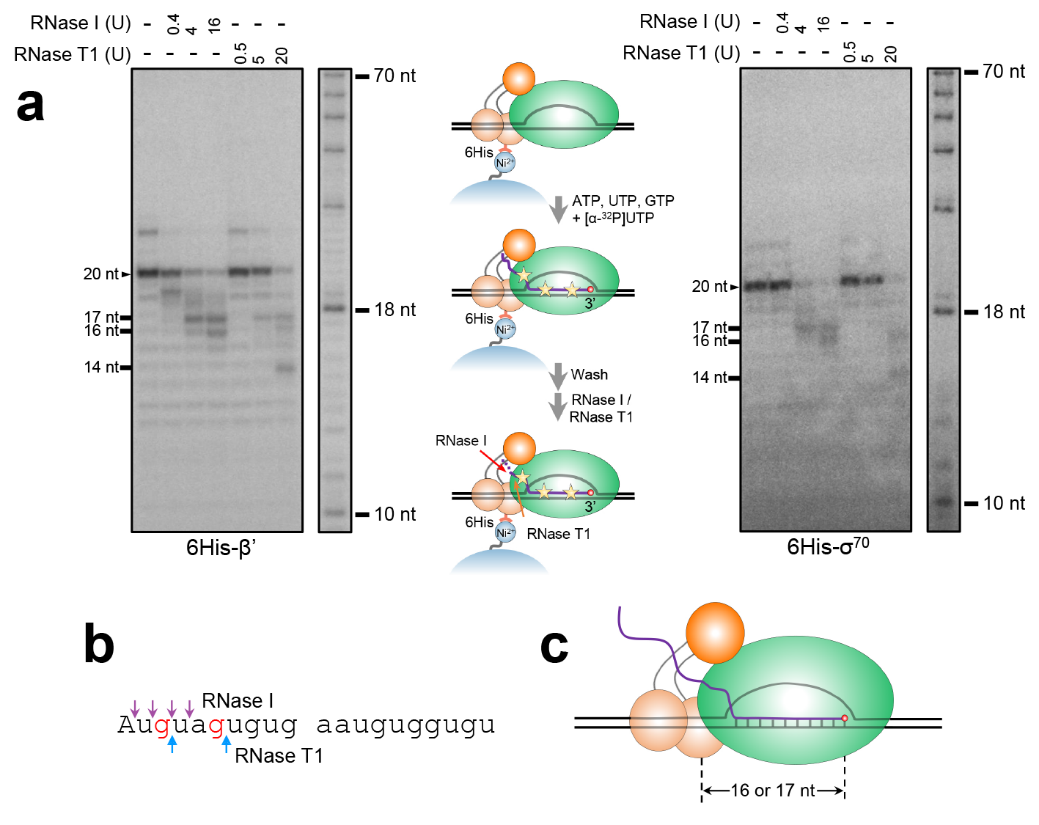


**Supplementary Fig. 2 In vitro analysis of Eσ^70^ holoenzyme footprints on the nascent RNA using TEC20 obtained from the *rrnB* P1 promoter and treated with RNase T1 and RNase I.** **a** Scheme in the middle shows the workflow of the experiment. The stars represent [α-^32^P] UMP incorporated into the nascent RNA. Red circle, RNA 3’ end. Eσ^70^ holoenzymes carrying a His-tag fused to β’ (left) or σ^70^ (right) was used to form the elongation complex carrying 20-nt RNA (TEC20) obtained from the *rrnB* P1 promoter in the presence of ATP, GTP, UTP and [α-^32^P] UTP, but without CTP. TEC20 was isolated on Ni^2+^-NTA beads by the β’ or the σ^70^ tag followed by treatment with the indicated amount of RNase I or RNase T1. Lanes on the right side of the gels display the RNA ladder. Data shown are representative of two independent experiments. **b** Sequence of the 20-nt nascent RNA and positions of the cleavage sites by RNase I (magenta arrows) and RNase T1 (blue arrows) are shown. **c** Schematic showing the length of nascent RNA in TEC20 protected by Eσ^70^.


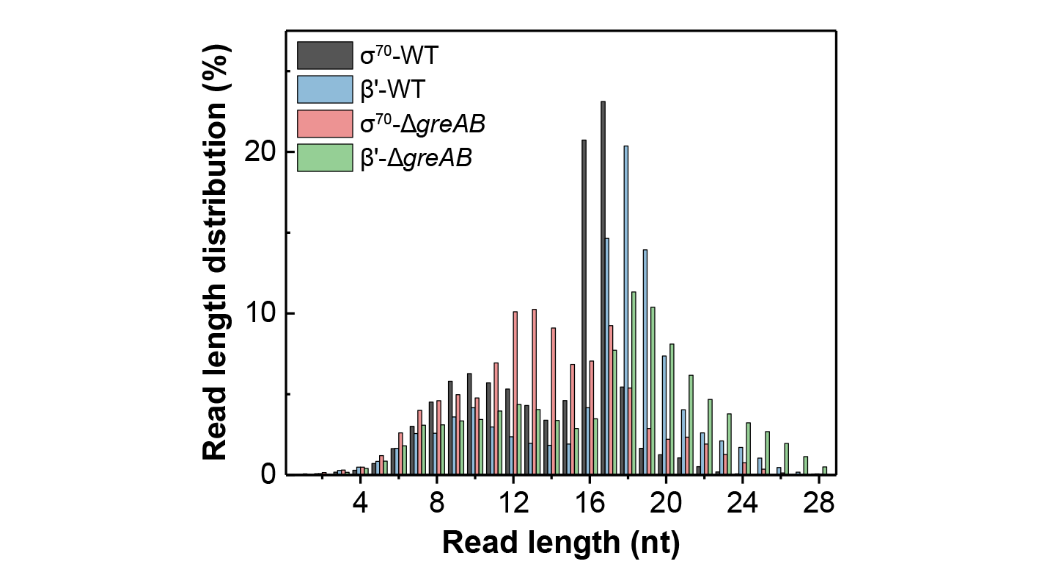


**Supplementary Fig. 3 The RNA length distribution in RNET-seq libraries from the indicated strains.** Histogram shows RNA length distributions for RNET-seq reads before alignment to the genome from the indicated strains. The average read lengths for σ^70^-WT, β’-WT, σ^70^-Δ*greAB* and β’-Δ*greAB* strains are 14.1-nt, 16.4-nt, 13.6-nt and 16.8-nt, respectively.


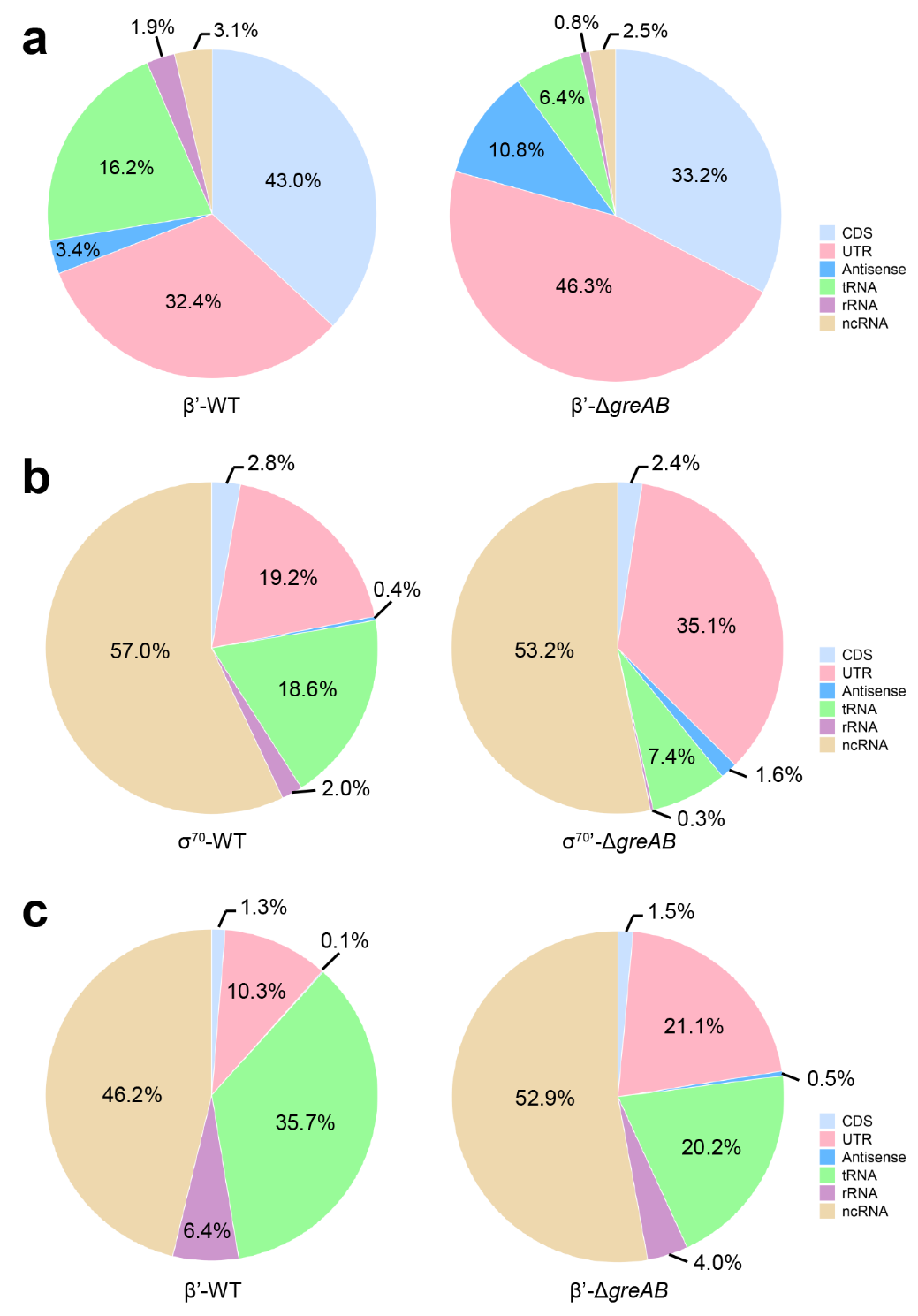


**Supplementary Fig. 4 Distribution of the pause sites and normalized enrichment across different genomic regions in different strains.** **a** Distribution of β’-dependent pauses among CDS, UTR, Antisense, tRNA, rRNA and ncRNA regions in β’-WT and β’-Δ*greAB* strains. β’-WT, *n* = 3543; β’-Δ*greAB*, *n* = 6498. The normalized distribution of σ^70^- **(b)** and β’- dependent **(c)** pause sites across different genomic regions. The lengths of all genomic regions are normalized to the same scale (per 1 kb).


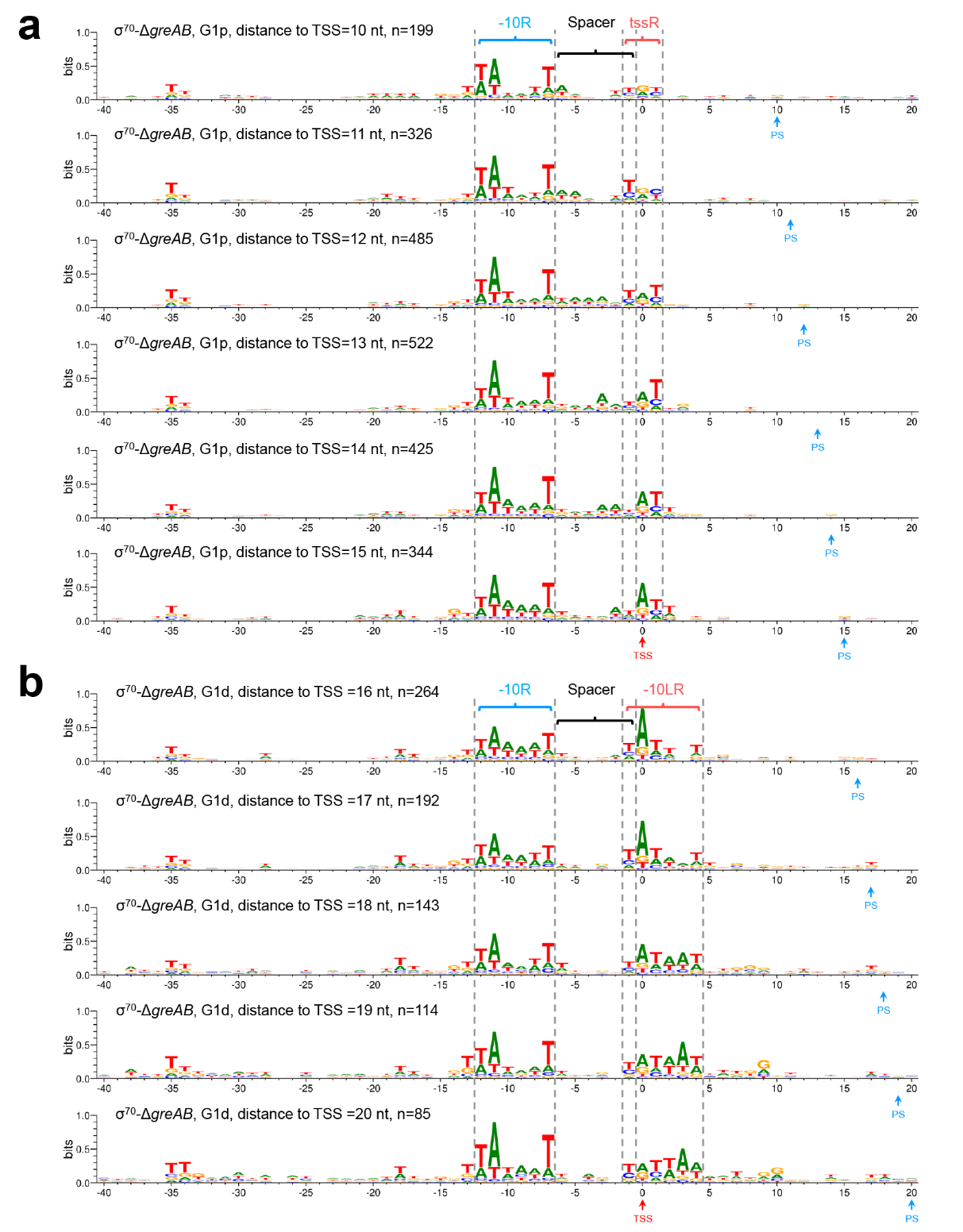


**Supplementary Fig. 5 Sequence logo analysis of G1 pause sites in σ^70^-Δ*greAB* cells located at the indicated distances from the TSS.** The DNA sequences surrounding the proximal G1p **(a)** and distal G1d **(b)** pause sites were sorted based on their distance from the TSS and aligned by centering at the corresponding TSS. In the sequence logo, “0” coordinate represents the TSS (red arrow). Blue arrow shows the 3’ RNA end at the pause sites (PS). The -10 element (-10R), spacer, TSS, and tssR/-10LR elements are indicated.


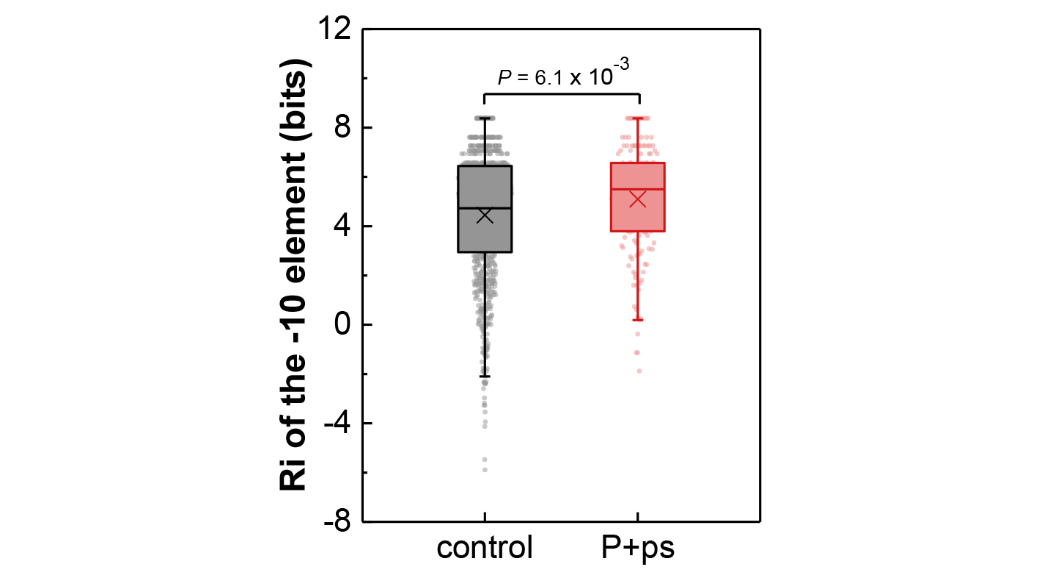


**Supplementary Fig. 6 Boxplot shows a comparison of Ri of -10R of control σ^70^ promoters and σ^70^ promoters containing σ^70^-dependent pauses.** control, all σ^70^ promoters from RegulonDB with a labeled -10 element (*n* = 950 promoters); P+ps, σ^70^ promoters with corresponding σ^70^-dependent pause sites identified in this study (*n* = 167 promoters). *P* value was calculated by two-tailed Mann-Whitney *U*-test. The median (solid line), mean (cross), 25th and 75th percentiles are indicated, and the whiskers represent 1.5-fold interquartile range.


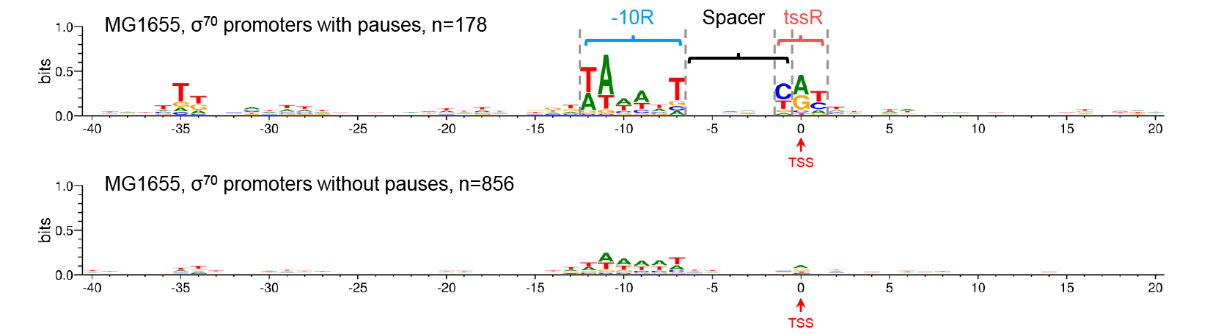


**Supplementary Fig. 7 Sequence logo analysis of the σ^70^ promoters with (top) and without (bottom) σ^70^-dependent pauses identified by RNET-seq.** Sequence logo of all σ^70^ promoters from RegulonDB except promoters that contain σ^70^-dependent pauses is shown at the bottom panel. DNA sequences were aligned relative to the corresponding TSS. In the sequence logo, coordinate “0” represents TSS.


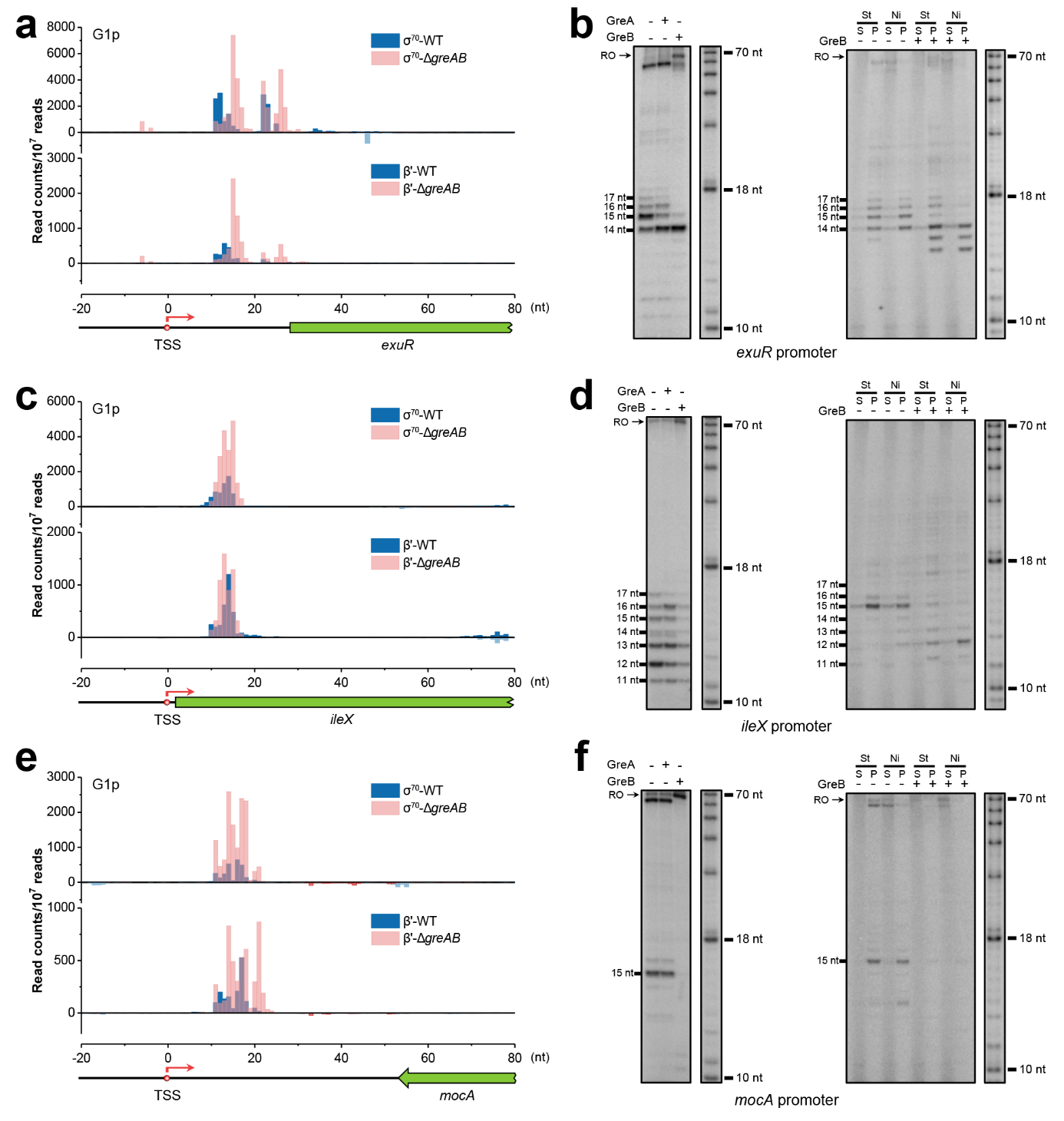


**Supplementary Fig. 8 In vitro validation of σ^70^-dependent G1p pauses.** RNET-seq data showing the σ^70^-dependent G1p pauses near *exuR* **(a)**, *ileX* **(c)** and *mocA* **(e)** genes. In vitro transcription assay confirmed the pause sites (left) and presence of σ^70^ in the paused complexes (right) obtained from *exuR* **(b)**, *ileX* **(d)** and antisense *mocA* **(f)** promoters. The left-side gels of **b, d, f** panels show the effect of GreA and GreB on the G1 pauses. The right-side gels of **b, d, f** panels show the RNAs pulled down on streptavidin beads by the 5’-biotinylated DNA templates (St), or by His-tag in σ^70^ subunit (Ni). S, supernatant fraction over the beads; P, RNAs retained on beads; RO, run-off transcript. Results represent two to four independent experiments.


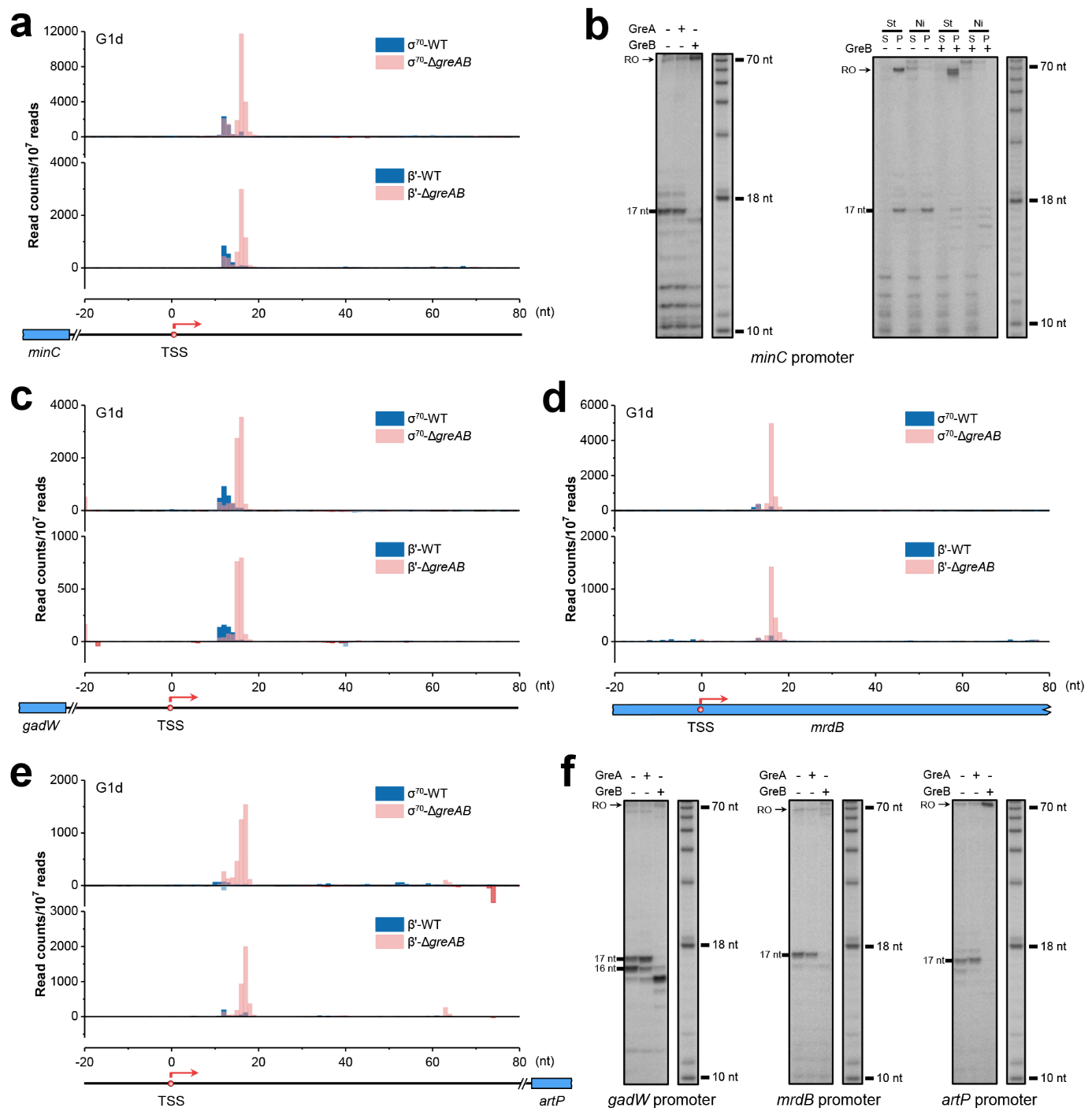


**Supplementary Fig. 9 In vitro validation of σ^70^-dependent G1d pauses.** The σ^70^-dependent backtracked G1d pauses were observed in RNET-seq data near or inside *minC* **(a)**, *gadW* **(c)**, *mrdB* **(d)** and *artP* **(e)** genes. In vitro transcription assay confirmed the pause site (left) and presence of σ^70^ in the paused complex (right) obtained from *minC* **(b)** promoter. In vitro transcription reproduced the σ^70^-dependent G1d pauses at the promoters near or inside *gadW, mrdB* and *artP* **(f)** genes. Results shown are representative of two to four independent experiments. See legend of Supplementary Fig. 8 for description of other experimental details.


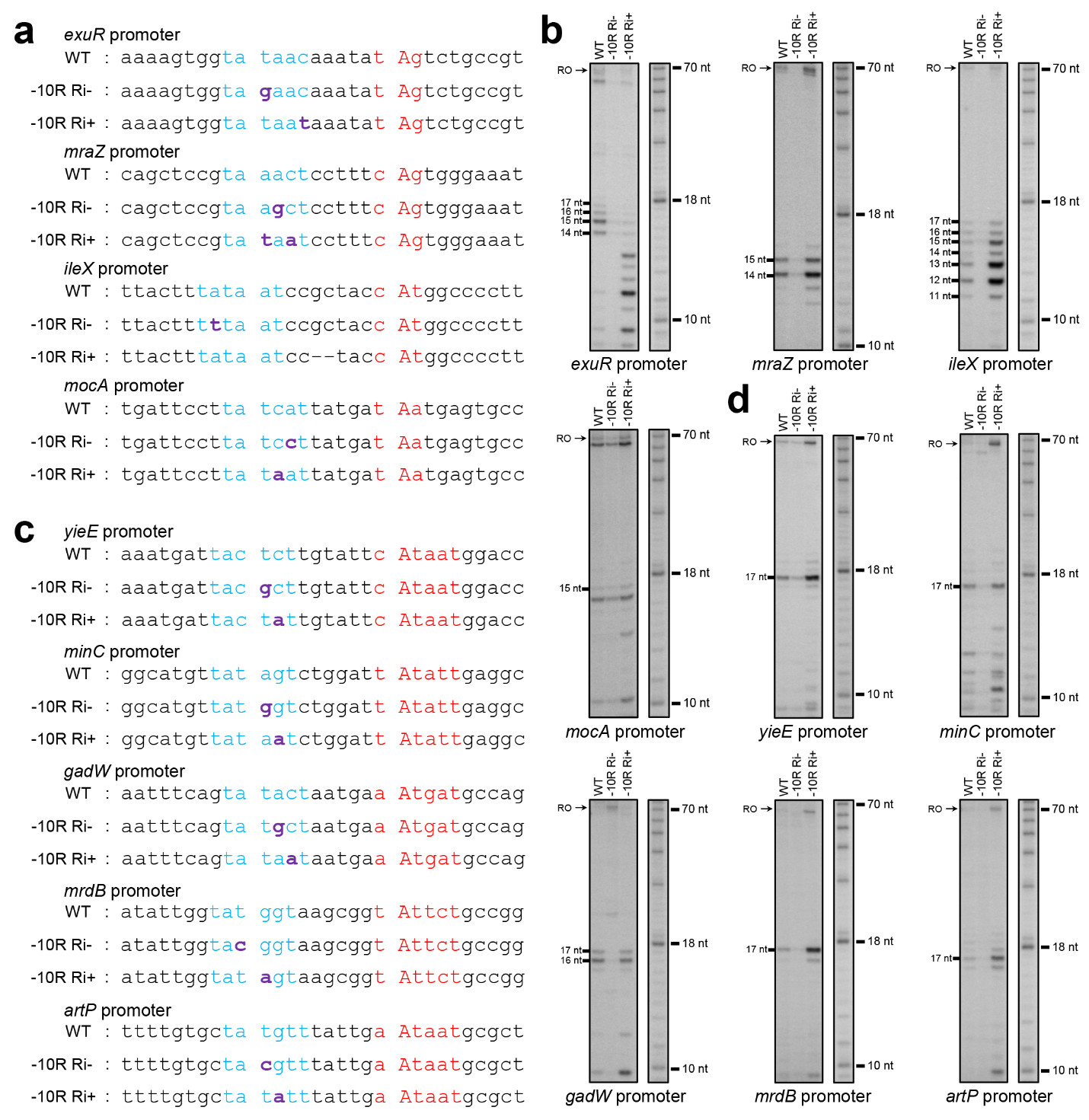


**Supplementary Fig. 10 Mutations in -10R promoter element affect G1p and G1d pauses in vitro.** The sequences of wild-type G1p/G1d promoters and the Ri-/+ mutations in -10R of the G1p **(a)** and G1d **(c)** promoters are shown. -10R is shown in blue; tssR/-10LR is shown in red; TSS is shown in red capital letter. The mutated positions are shown in purple. In vitro transcription reveals the effect of the -10R mutations on pause strength at both G1p **(b)** and G1d **(d)** promoters. -10R Ri-/+, mutated -10R with decreased/ increased Ri. Data shown represent three independent experiments.


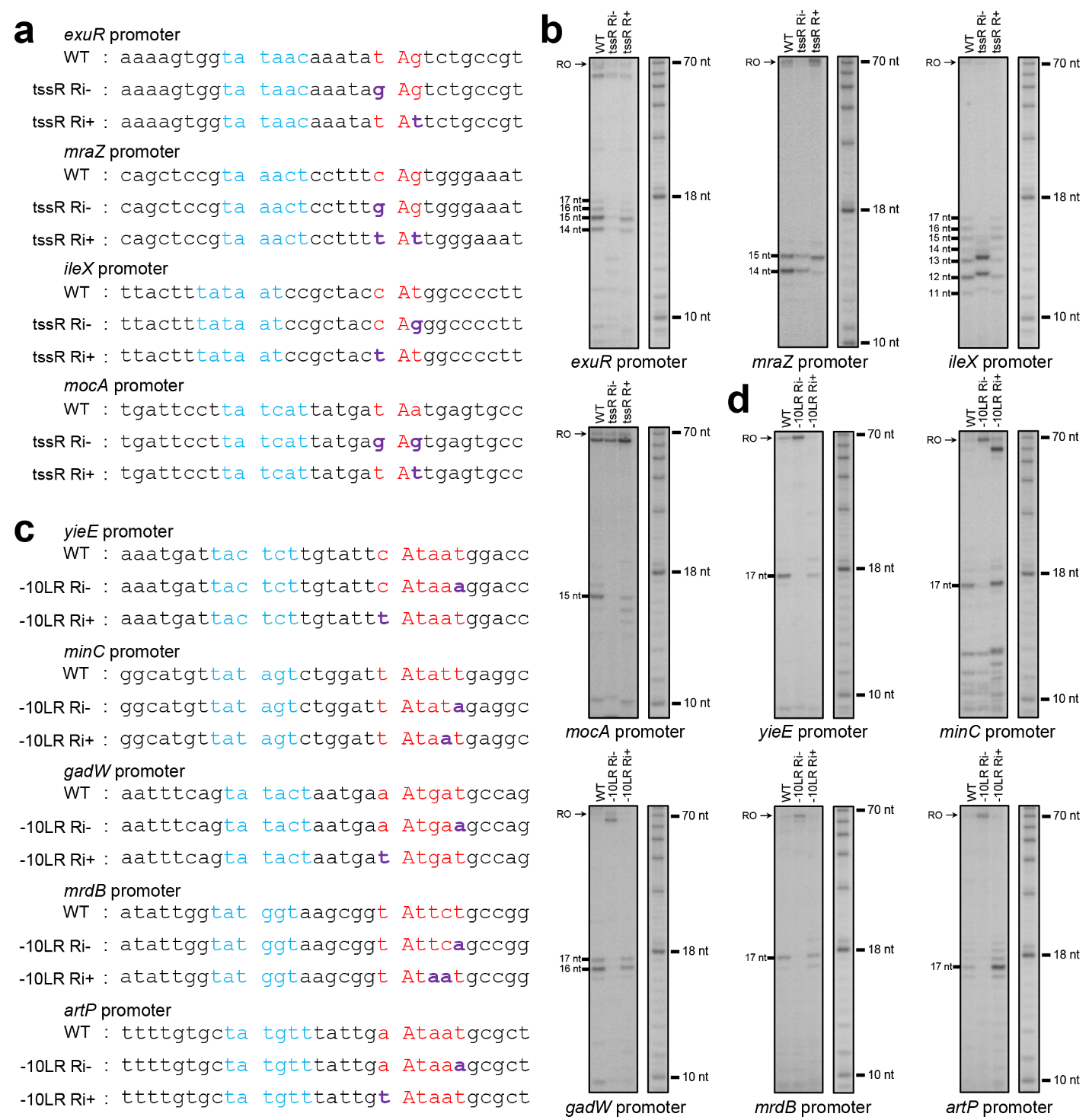


**Supplementary Fig. 11 tssR and -10LR elements on G1p and G1d promoters dictate the pause strength.** The DNA sequences of wild type, tssR Ri-/+ mutations of G1p **(a)** promoters and -10LR Ri-/+ mutations of G1d **(c)** promoters are shown. -10R is shown in blue; tssR/-10LR is shown in red; TSS is shown in red capital letter. In vitro transcription confirms importance of the tssR and -10LR sequences on pause strength at G1p **(b)** and G1d **(d)** promoter, respectively. tssR Ri-/+, mutated tssR with decreased/increased Ri; -10LR Ri-/+, mutated -10LR with decreased/increased Ri. Short abortive transcripts ~10 nt accumulate during in vitro transcription on some (but not all) promoters. Data shown are representative of three independent experiments.


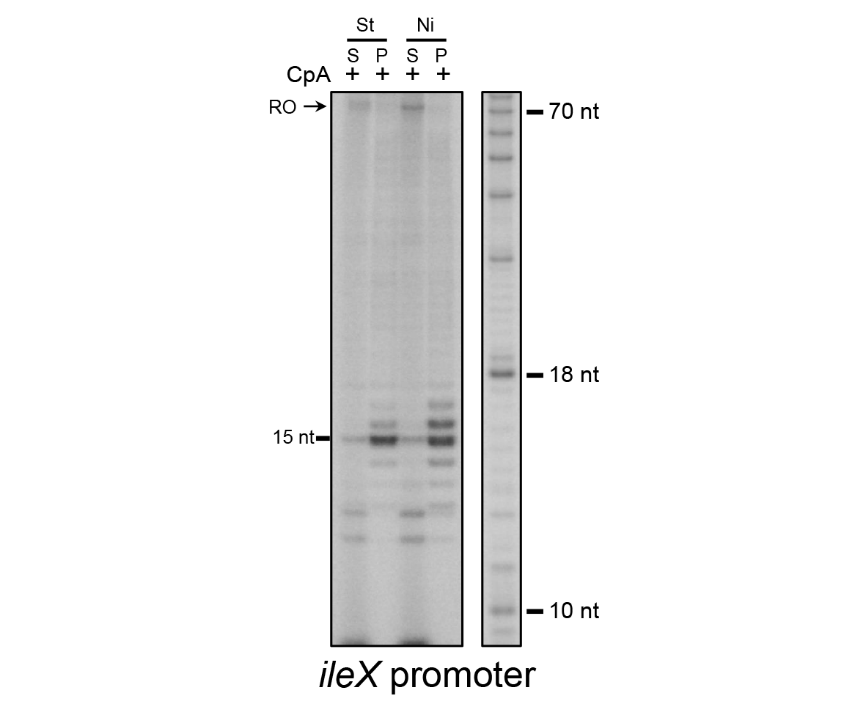


**Supplementary Fig. 12 G1 pausing in dinucleotide-primed in vitro transcription is σ^70^-dependent.** 5’-biotinylated *ileX* promoter DNA and Eσ^70^ holoenzyme with the His-tagged σ^70^ subunit were used for in vitro transcription with CpA_+1_ dinucleotide as the RNA primer. This primer shifted TSS 1 bp upstream from the original A_+1_ position of *ileX* promoter. Data are representative of two independent experiments. See legend of Supplementary Fig. 8 for description of other experimental details.


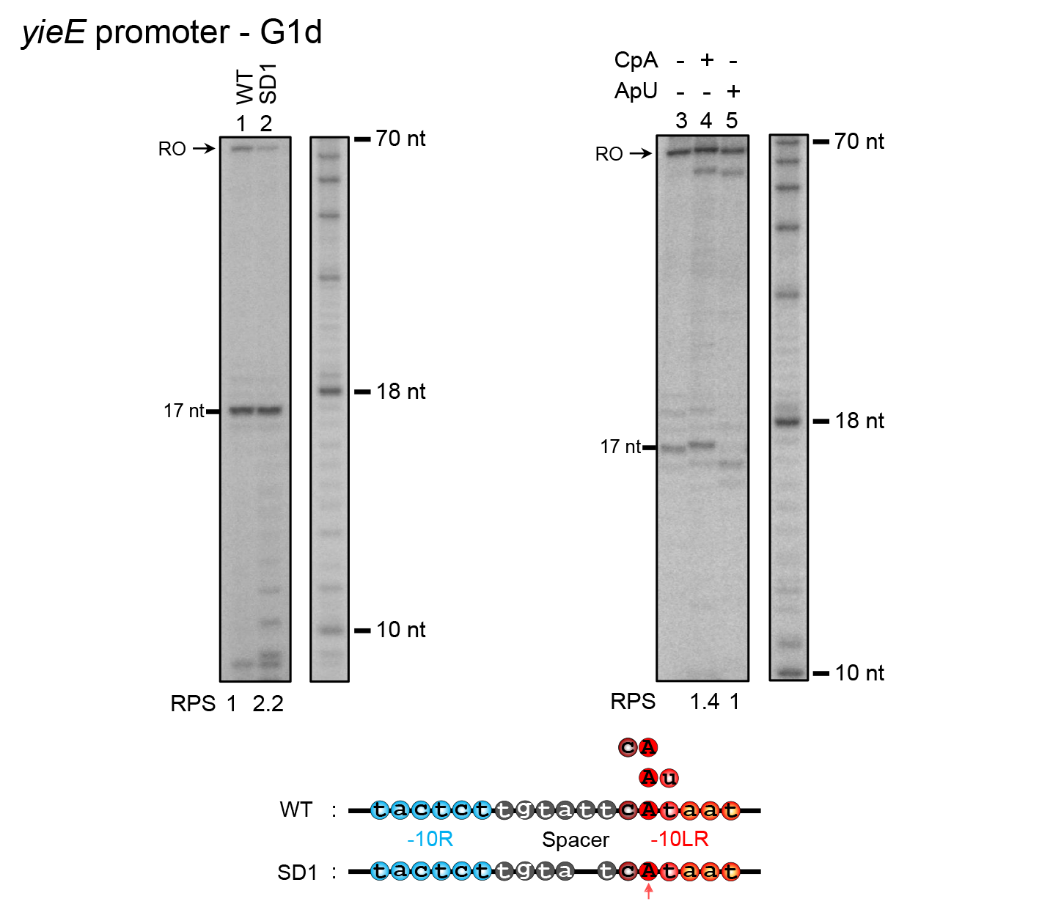


**Supplementary Fig. 13 Strength of G1d pause at *yieE* promoter depends on the spacer length.** In vitro transcription on wild type (lane 1, WT) or mutant (lane 2, SD1) DNA template (left-side panel). The G1d pause strength varies depending on RNA primer used for initiation of transcription on wild-type *yieE* promoter. Positions and sequences of the A_+1_pU primer for the natural TSS, and CpA_+1_ primer are shown. CpA_+1_ primer shifts TSS 1 nt closer to -10R. WT, wild-type promoter; SD1; 1-bp shortened spacer; RPS, relative pause strength. The representative results are shown based on two to three independent experiments.


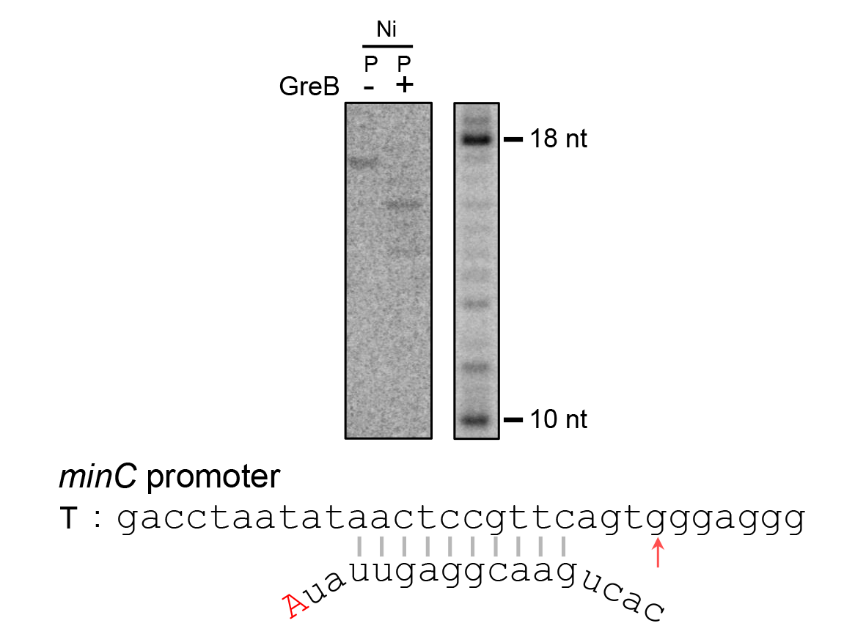


**Supplementary Fig. 14 GreB-induced transcript cleavage of the nascent RNA at the G1d*^minC^* pause.** Sequence of the template DNA strand (T) from the *minC* promoter and the backtracked RNA at the G1d*^minC^* pause are shown at the bottom. Red arrow, 3’ RNA end at the pause. The capital A letter, 5’ RNA end. Results are representative of two independent experiments. See legend of Supplementary Fig. 8 for description of other experimental details.


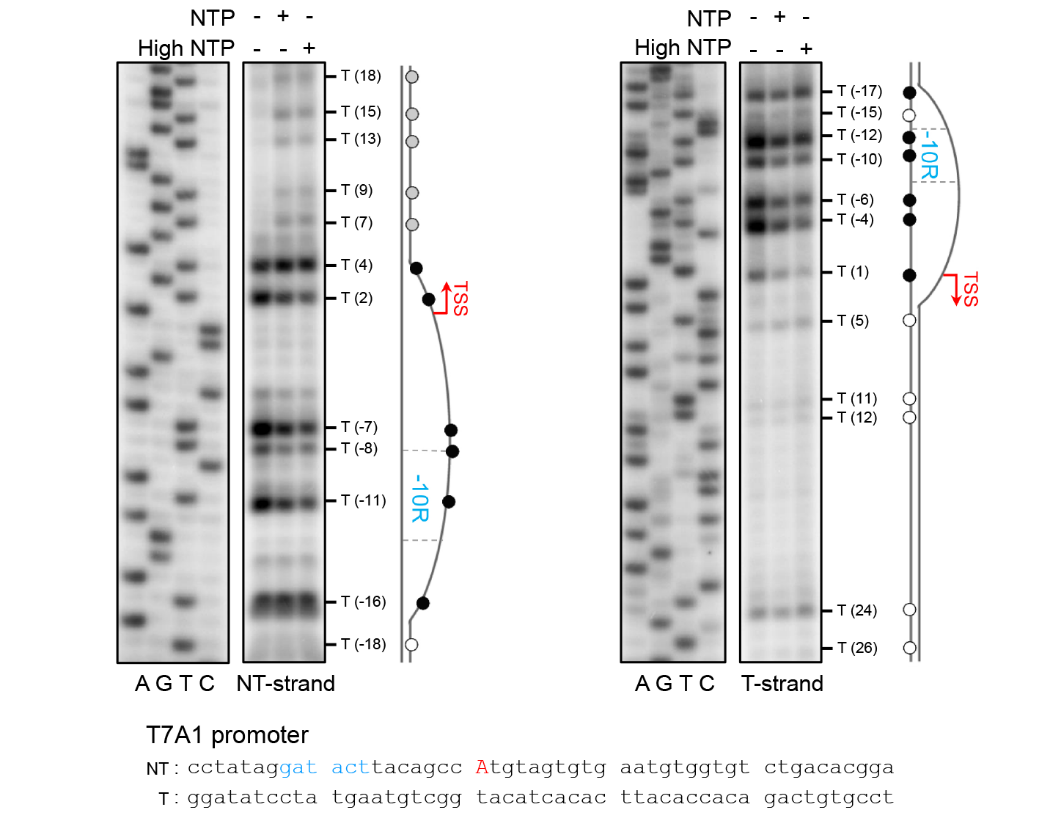


**Supplementary Fig. 15 Potassium permanganate footprinting of the template and non-template DNA strands in transcription bubble at the T7A1 promoter.** The positions of all T residues in the bubble are indicated. The diagrams on the right show the transcription bubble in T7A1 RPo and its conversion into a TEC by incubation with 1 mM or 20 μM NTPs as indicated. Black filled circles, T residues sensitive to KMnO_4_ in the absence and presence of NTP; gray filled circles, permanganate-sensitive T residues only in the presence of NTP; white filled circle, T residues resistant to permanganate. Results are representative of two independent experiments.


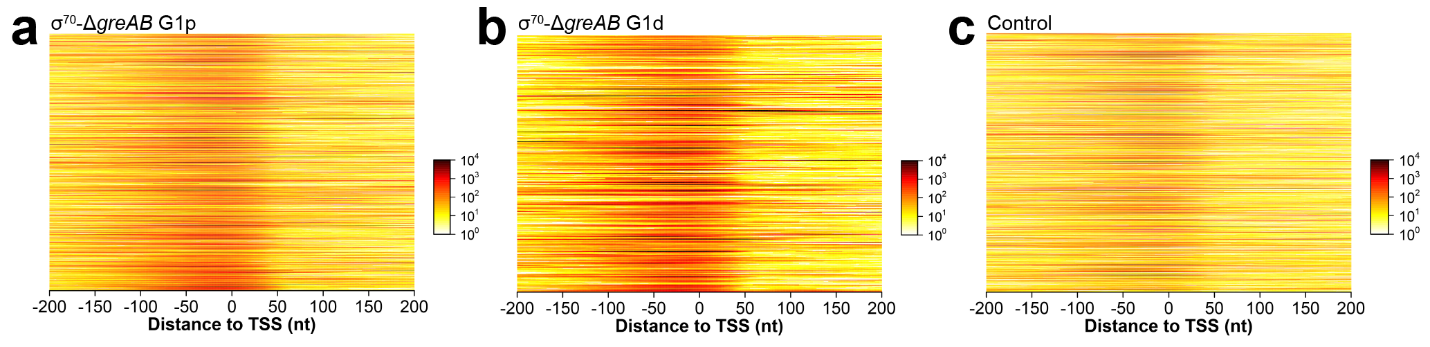


**Supplementary Fig. 16 Heatmaps showing the reads coverage from ChIP-seq data at G1p and G1d promoters.** The normalized read coverage surrounding the TSS (± 200 nt) of σ^70^-Δ*greAB* G1p (*n* = 1069) and G1d (*n* = 407) promoters were shown. The randomly selected promoters lacking G1 pauses were used as a control (*n* = 1299). ChIP-seq data were obtained from reference^1^.


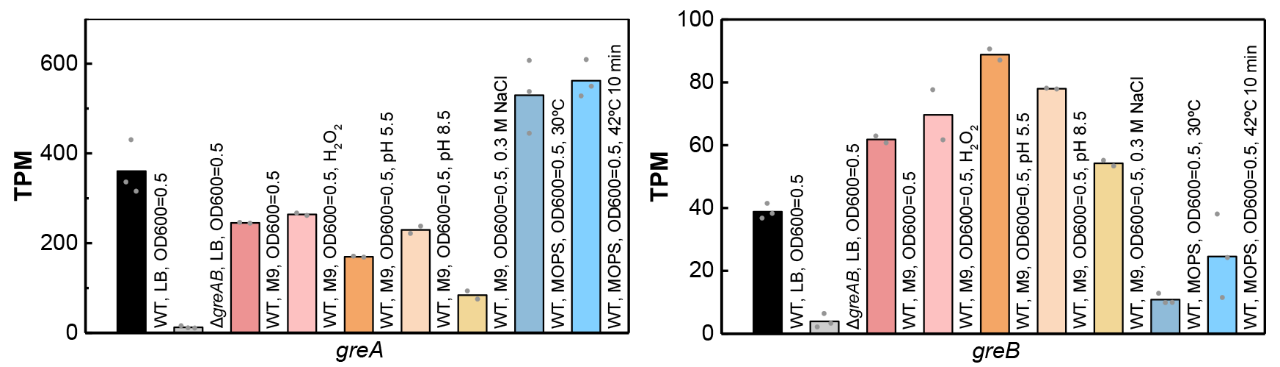


**Supplementary Fig. 17 Expression of *greA* and *greB* genes in transcripts per million (TPM) from RNA-seq data at different growth conditions.** The RNA-seq data were taken from GEO with the following accession numbers GSE135516^2^, GSE111094^3^, GSE88980^4^ and GSE90056. The labels at the right of each column display the strain identity, growth medium, OD_600_ used to collect the sample, and a special treatment of the cells when indicated. The analysis included *n* = 3, 3, 2, 2, 2, 2, 2, 3 and 3 biological replicates for each column from left to right, respectively.


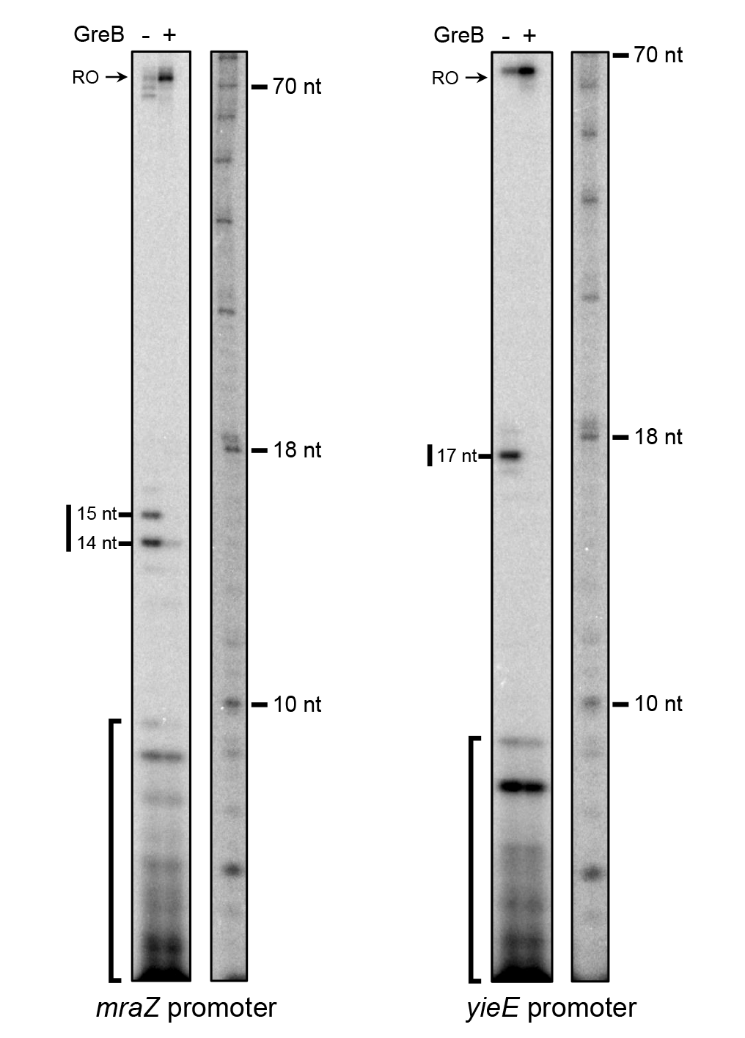


**Supplementary Fig. 18 Effect of GreB on the σ^70^-dependent G1p/G1d pauses and abortive transcription during initiation at G1 promoters in vitro.** In vitro transcription assay was performed using *mraZ* (G1p) and *yieE* (G1d) promoters in the absence or presence of GreB. RO, run-off transcript; vertical bar, paused transcripts; vertical brackets, abortive transcripts. The representative results are based on three independent experiments.

**Supplementary references**

1. Singh, S. S. et al. Widespread suppression of intragenic transcription initiation by H-NS. *Genes Dev.* **28**, 214-219 (2014).

2. Anand, A. et al. OxyR is a convergent target for mutations acquired during adaptation to oxidative stress-prone metabolic states. *Mol. Biol. Evol.* **37**, 660-667 (2020).

3. Gao, Y. et al. Systematic discovery of uncharacterized transcription factors in *Escherichia coli* K-12 MG1655. *Nucleic Acids Res.* **46**, 10682-10696 (2018).

4. Seo, S. W. et al. Revealing genome-scale transcriptional regulatory landscape of OmpR highlights its expanded regulatory roles under osmotic stress in *Escherichia coli* K-12 MG1655. *Sci. Rep.* **7**, 2181 (2017).
