# Supplementary material for: Nascent RNA sequencing identifies a widespread sigma70-dependent pausing regulated by Gre factors in bacteria
Source: Nat Commun. 2021 Feb 10;12:906. doi: 10.1038/s41467-021-21150-2 (PMC7876045; doi:10.1038/s41467-021-21150-2)
Supplement: Supplementary file 3 — Reporting Summary [file 41467_2021_21150_MOESM3_ESM.pdf]

nature research

Corresponding author(s): Mikhail Kashlev

Last updated by author(s): Jan 11, 2021

# Reporting Summary

Nature Research wishes to improve the reproducibility of the work that we publish. This form provides structure for consistency and transparency in reporting. For further information on Nature Research policies, see our Editorial Policies and the Editorial Policy Checklist.

## Statistics

For all statistical analyses, confirm that the following items are present in the figure legend, table legend, main text, or Methods section.

| n/a | Confirmed | |
|---|---|---|
| ☐ | ☒ | The exact sample size (*n*) for each experimental group/condition, given as a discrete number and unit of measurement |
| ☐ | ☒ | A statement on whether measurements were taken from distinct samples or whether the same sample was measured repeatedly |
| ☐ | ☒ | The statistical test(s) used AND whether they are one- or two-sided<br>*Only common tests should be described solely by name; describe more complex techniques in the Methods section.* |
| ☒ | ☐ | A description of all covariates tested |
| ☒ | ☐ | A description of any assumptions or corrections, such as tests of normality and adjustment for multiple comparisons |
| ☐ | ☒ | A full description of the statistical parameters including central tendency (e.g. means) or other basic estimates (e.g. regression coefficient) AND variation (e.g. standard deviation) or associated estimates of uncertainty (e.g. confidence intervals) |
| ☐ | ☒ | For null hypothesis testing, the test statistic (e.g. $F$, $t$, $r$) with confidence intervals, effect sizes, degrees of freedom and $P$ value noted<br>*Give P values as exact values whenever suitable.* |
| ☒ | ☐ | For Bayesian analysis, information on the choice of priors and Markov chain Monte Carlo settings |
| ☒ | ☐ | For hierarchical and complex designs, identification of the appropriate level for tests and full reporting of outcomes |
| ☐ | ☒ | Estimates of effect sizes (e.g. Cohen's $d$, Pearson's $r$), indicating how they were calculated |

*Our web collection on statistics for biologists contains articles on many of the points above.*

## Software and code

Policy information about availability of computer code

Data collection

RNET-seq raw data were collected on Illumina HiSeq 2500 by the NIH Intramural Sequencing Center.
RNA-seq raw data were collected on MiSeq by the Center for Cancer Research Sequencing Facility.
Basecalling was performed by RTA 1.18.
Adaptor and barcode were removed by cutadapt 1.18.

Data analysis

For RNET-seq, duplicates were removed by bbmap 38.22.
RNET-seq reads were aligned to E. coli MG1655 genome NC_000913.2 using bowtie 1.2.2.
RNA-seq reads were mapped to the genome by STAR 2.6.1.
For RNET-seq, the 5’ end coordinates of all uniquely aligned R1 reads were recorded and the total read counts at each coordinate were determined by bedtools 2.27.1.
For RNA-seq, the raw counts of aligned reads in each gene were calculated by HTseq 0.11.2.
Fold changes of genes transcription between different samples were calculated by DESeq2 1.26.0.
Pause peaks were determined by custom R scripts.
Custom scripts were available at https://github.com/Mikhail-NCI-Lab/RNET-seq_code.

For manuscripts utilizing custom algorithms or software that are central to the research but not yet described in published literature, software must be made available to editors and reviewers. We strongly encourage code deposition in a community repository (e.g. GitHub). See the Nature Research guidelines for submitting code & software for further information.

## Data

Policy information about availability of data

All manuscripts must include a data availability statement. This statement should provide the following information, where applicable:

- Accession codes, unique identifiers, or web links for publicly available datasets
- A list of figures that have associated raw data
- A description of any restrictions on data availability

All RNET-seq and RNA-seq data from this study were deposited to NCBI's Gene Expression Omnibus (GEO) database (https://www.ncbi.nlm.nih.gov/geo) under the accession number GSE147611. The RNA-seq data used for greA and greB genes expression were obtained from GEO with the accession numbers GSE135516, GSE111094, GSE88980 and GSE90056. All uncropped gel images are included in source data files. Source data for all figures are provided with the paper.

# Field-specific reporting

Please select the one below that is the best fit for your research. If you are not sure, read the appropriate sections before making your selection.

☒ Life sciences ☐ Behavioural & social sciences ☐ Ecological, evolutionary & environmental sciences

For a reference copy of the document with all sections, see nature.com/documents/nr-reporting-summary-flat.pdf

# Life sciences study design

All studies must disclose on these points even when the disclosure is negative.

| | |
|---|---|
| Sample size | No statistical methods were used to predetermine sample sizes. Each experiment was independently performed two or three times, because of the high sequencing depth (> 9 M reads/RNET-seq library; > 2 M reads/RNA-seq library), the small E. coli genome (4.64 M) and the high reproducibility (high correlation between two biological replicates). |
| Data exclusions | RNET-seq reads not uniquely aligned to the genome were discarded and not used for analysis. |
| Replication | Two independent biological replicates of RNET-seq libraries and three independent biological replicates of RNA-seq libraries were made and high reproducibility of the data was confirmed. All biochemical experiments were reliably reproduced after being repeated two or three times. |
| Randomization | Randomization is not applicable since samples were not allocated into group. |
| Blinding | Blinding is not applicable since samples were not allocated into group. |

# Reporting for specific materials, systems and methods

We require information from authors about some types of materials, experimental systems and methods used in many studies. Here, indicate whether each material, system or method listed is relevant to your study. If you are not sure if a list item applies to your research, read the appropriate section before selecting a response.

### Materials & experimental systems

| n/a | Involved in the study |
|---|---|
| ☒ | ☐ Antibodies |
| ☒ | ☐ Eukaryotic cell lines |
| ☒ | ☐ Palaeontology and archaeology |
| ☒ | ☐ Animals and other organisms |
| ☒ | ☐ Human research participants |
| ☒ | ☐ Clinical data |
| ☒ | ☐ Dual use research of concern |

### Methods

| n/a | Involved in the study |
|---|---|
| ☒ | ☐ ChIP-seq |
| ☒ | ☐ Flow cytometry |
| ☒ | ☐ MRI-based neuroimaging |
