# Supplementary material for: Nascent RNA sequencing identifies a widespread sigma70-dependent pausing regulated by Gre factors in bacteria
Source: Nat Commun. 2021 Feb 10;12:906. doi: 10.1038/s41467-021-21150-2 (PMC7876045; doi:10.1038/s41467-021-21150-2)
Supplement: Supplementary file 4 — Description of Additional Supplementary Files [file 41467_2021_21150_MOESM4_ESM.pdf]

Description of Additional Supplementary Files

Supplementary Data 1: Pause sites identified by σ70- and β’-affinity in wild-type and ΔgreAB cells.

Supplementary Data 2: G1p and G1d pause sites identified by σ70-affinity in ΔgreAB cells.

Supplementary Data 3: Primers used in this study.
