# Supplementary material for: Nascent RNA sequencing identifies a widespread sigma70-dependent pausing regulated by Gre factors in bacteria
Source: Nat Commun. 2021 Feb 10;12:906. doi: 10.1038/s41467-021-21150-2 (PMC7876045; doi:10.1038/s41467-021-21150-2)

Summary

Fig. 1b, uncropped gel image

Fig. 3g, uncropped gel image
Fig. 3h, uncropped gel image

Fig. 4g, uncropped gel image
Fig. 4h, uncropped gel image

Fig. 5a, uncropped gel image
Fig. 5b, uncropped gel image
Fig. 5c, uncropped gel image
Fig. 5d, uncropped gel image
Fig. 5e, uncropped gel image

Fig. S2a, uncropped gel image

Fig. S8b, uncropped gel image
Fig. S8d, uncropped gel image
Fig. S8f, uncropped gel image

Fig. S9b, uncropped gel image
Fig. S9f, uncropped gel image

Fig. S10b, uncropped gel image
Fig. S10d, uncropped gel image

Fig. S11b, uncropped gel image
Fig. S11d, uncropped gel image

Fig. S12, uncropped gel image

Fig. S13, uncropped gel image

Fig. S14, uncropped gel image

Fig. S15, uncropped gel image

Fig. S18, uncropped gel image

Fig. 1b

Strain	1	2	3	1	2	3
GreB (5 µM)	-	-	-	+	+	+
Lane	1	2	3	4	5	6

1: WT (no His-tag)
2: β’-WT
3: σ^{70}-WT

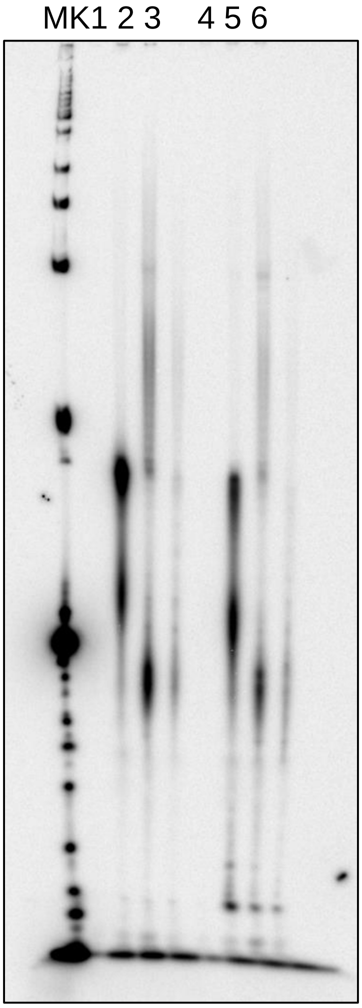

Fig. 3g

GreA	-	+	-
GreB	-	-	+
Lane	1	2	3

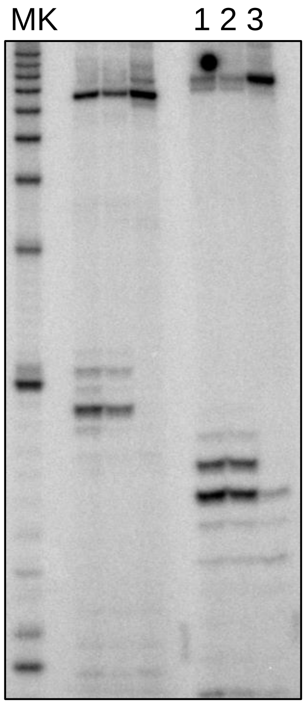

	St		Ni		St		Ni	
	S	P	S	P	S	P	S	P
GreB	-	-	-	-	+	+	+	+
Lane	1	2	3	4	5	6	7	8

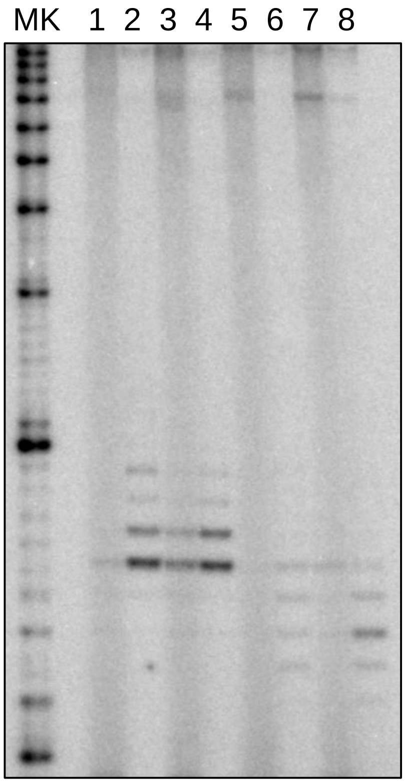

Fig. 3h

GreA	-	+	-
GreB	-	-	+
Lane	1	2	3

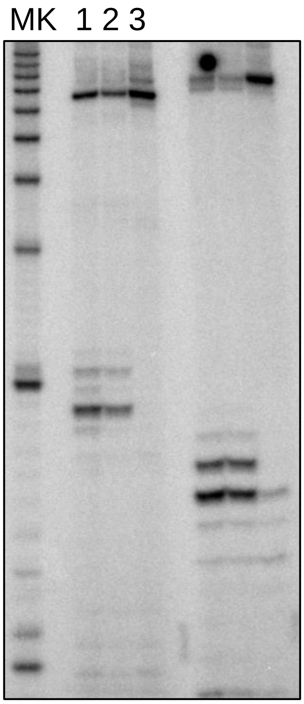

	St		Ni		St		Ni	
	S	P	S	P	S	P	S	P
GreB	-	-	-	-	+	+	+	+
Lane	1	2	3	4	5	6	7	8

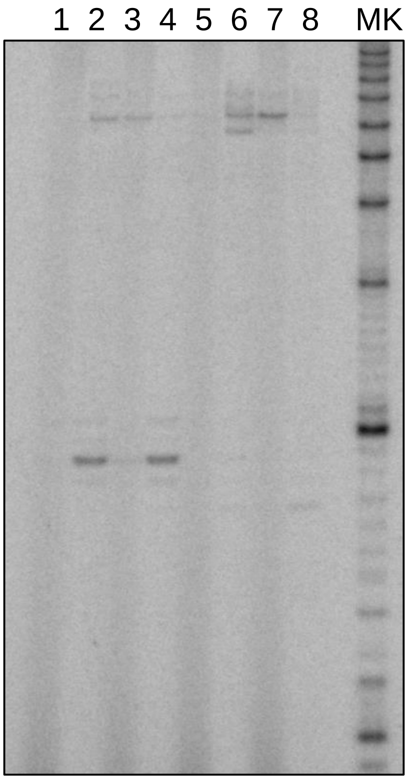

Fig. 4g

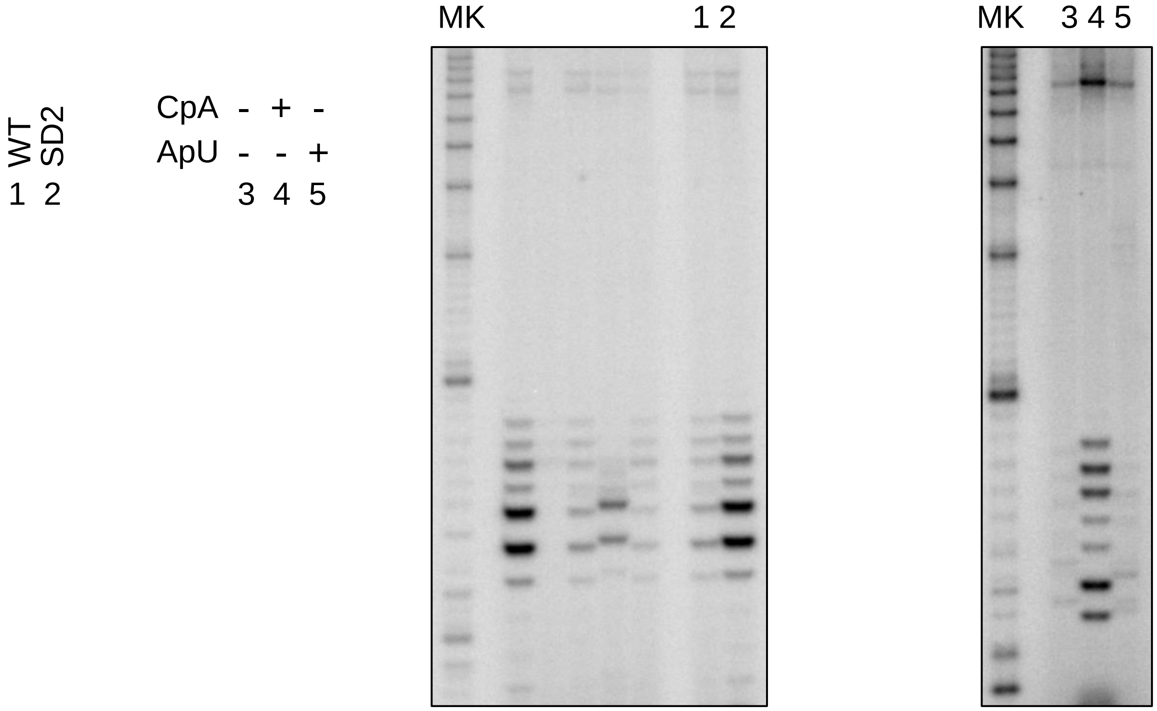
WT
SD2
1 2
CpA - + -
ApU - - +
3 4 5
MK
1 2
MK
3 4 5

Fig. 4h

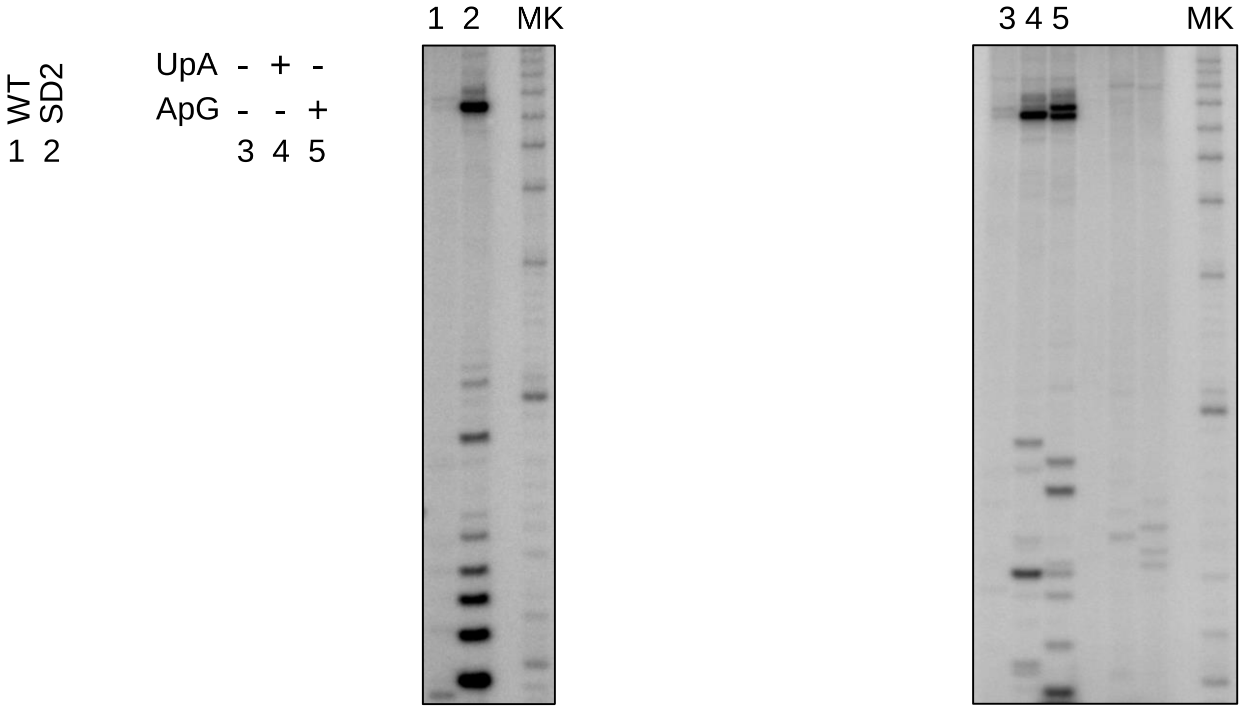
WT
SD2
1 2
UpA - + -
ApG - - +
3 4 5
1 2
MK
3 4 5
MK

Fig. 5a

RNase I (U)	-	0.4	4	16	-	-	-
RNase T1 (U)	-	-	-	-	0.5	5	20
Lane	1	2	3	4	5	6	7

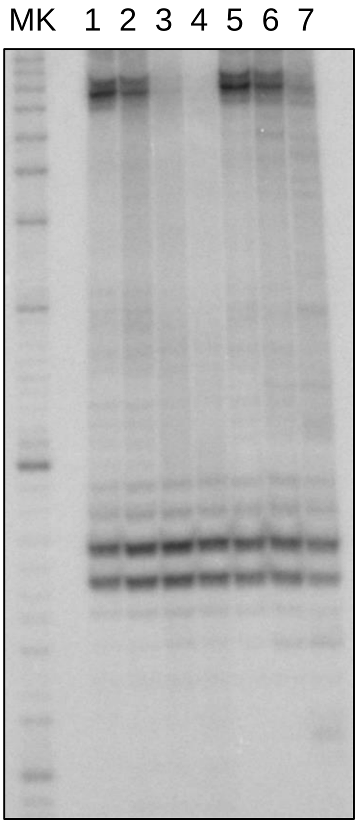

Fig. 5b

RNase I (U)	-	0.4	4	16	-	-	-
RNase T1 (U)	-	-	-	-	0.5	5	20
Lane	1	2	3	4	5	6	7

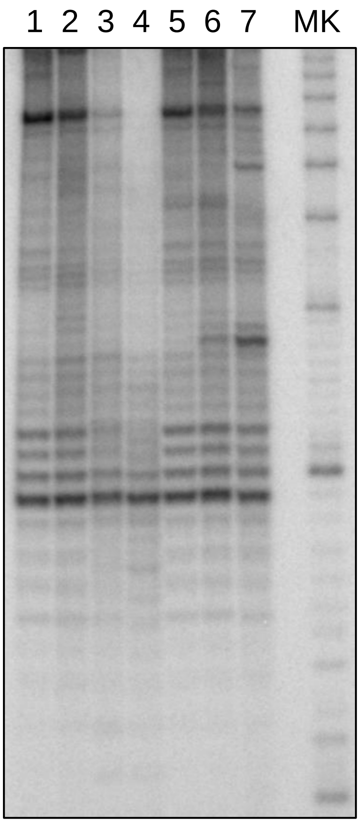

Fig. 5c

Fig. 5c (left)

	Ni P	Ni P
GreB	-	+
Lane	1	2

Fig. 5c (right)

	Ni P	Ni P
GreB	-	+
Lane	3	4

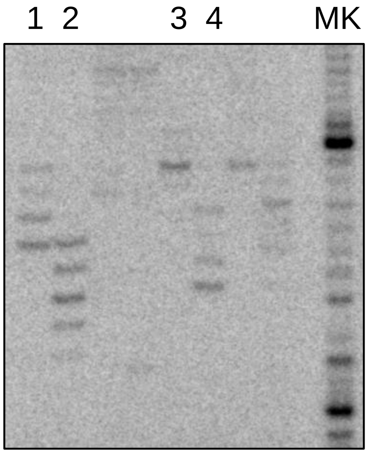

Fig. 5d

Fig. 5d (left)

Eσ70	-	+	+	+
NTP	-	-	+	+
GreB	-	-	-	+
Lane	1	2	3	4

Fig. 5d (right)

Eσ70	-	+	+	+
NTP	-	-	+	+
GreB	-	-	-	+
Lane	5	6	7	8

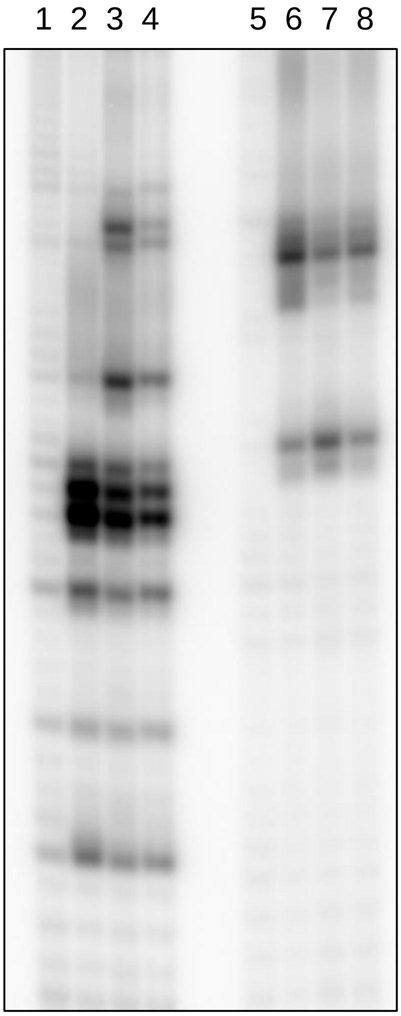

Fig. 5e

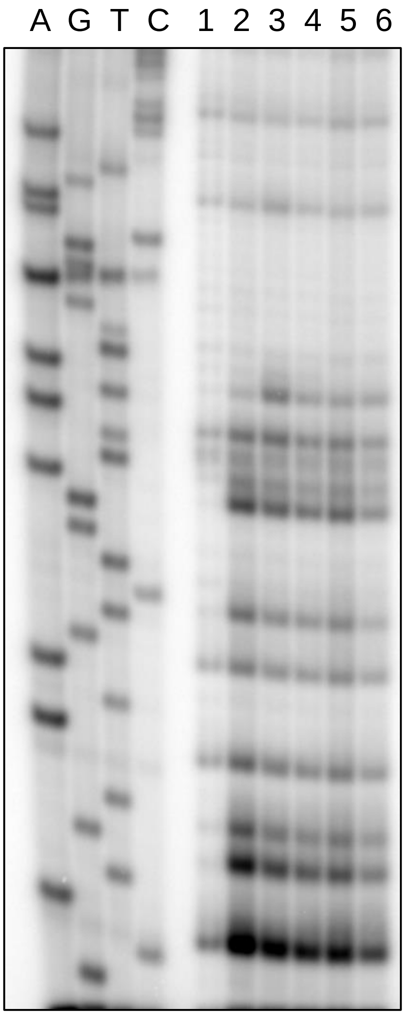

Eσ[70]	-	+	+	+	+	+
WT	+	+	+	+	-	-
-10LR Ri-	-	-	-	-	+	+
NTP	-	-	+	+	-	+
GreB	-	-	-	+	-	-
Lane	1	2	3	4	5	6

Fig. 5e (left)

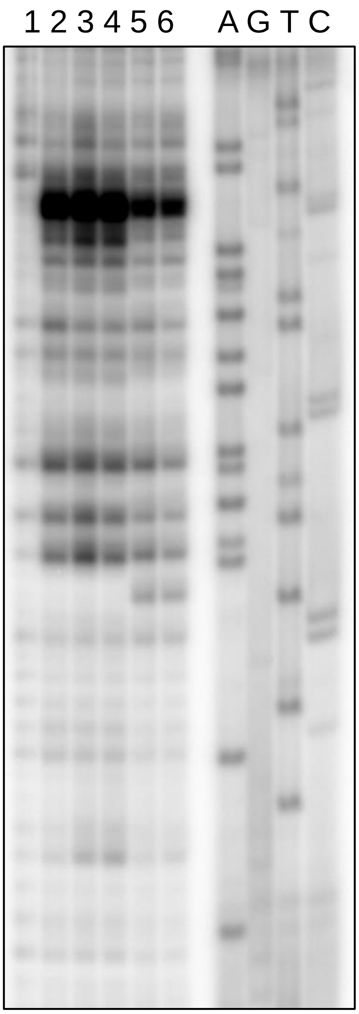

Eσ[70]	-	+	+	+	+	+
WT	+	+	+	+	-	-
-10LR Ri-	-	-	-	-	+	+
NTP	-	-	+	+	-	+
GreB	-	-	-	+	-	-
Lane	1	2	3	4	5	6

Fig. 5e (right)

Fig. S2a

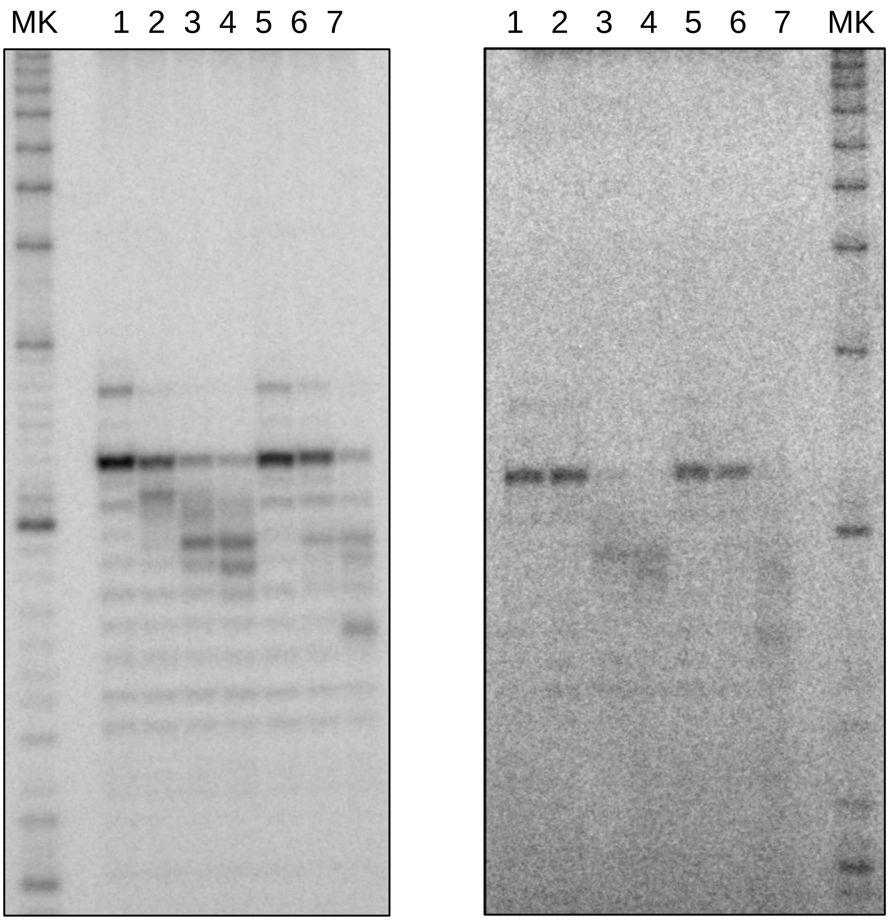

RNase I (U)	-	0.4	4	16	-	-	-
RNase T1 (U)	-	-	-	-	0.5	5	20
Lane	1	2	3	4	5	6	7

Fig. S2a (left)

RNase I (U)	-	0.4	4	16	-	-	-
RNase T1 (U)	-	-	-	-	0.5	5	20
Lane	1	2	3	4	5	6	7

Fig. S2a (right)

Fig. S8b

GreA	-	+	-
GreB	-	-	+
Lane	1	2	3

	St		Ni		St		Ni	
	S	P	S	P	S	P	S	P
GreB	-	-	-	-	+	+	+	+
Lane	1	2	3	4	5	6	7	8

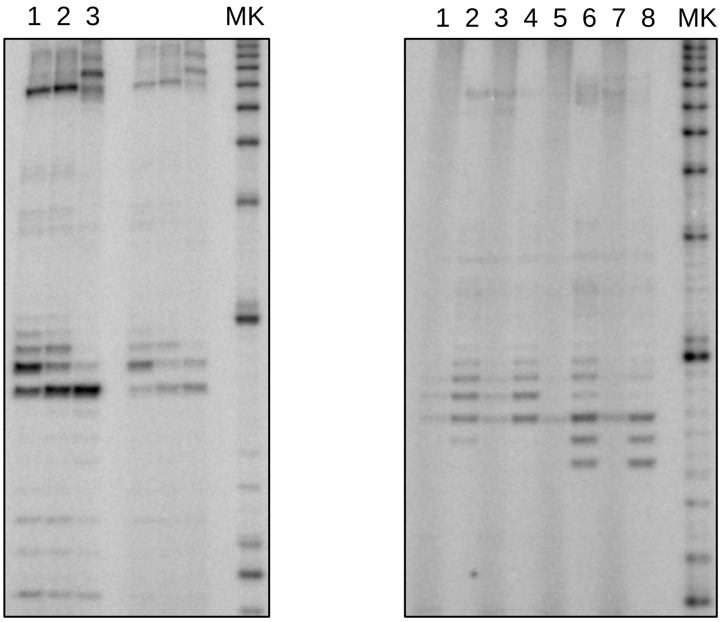

Fig. S8d

GreA	-	+	-
GreB	-	-	+
Lane	1	2	3

	St		Ni		St		Ni	
	S	P	S	P	S	P	S	P
GreB	-	-	-	-	+	+	+	+
Lane	1	2	3	4	5	6	7	8

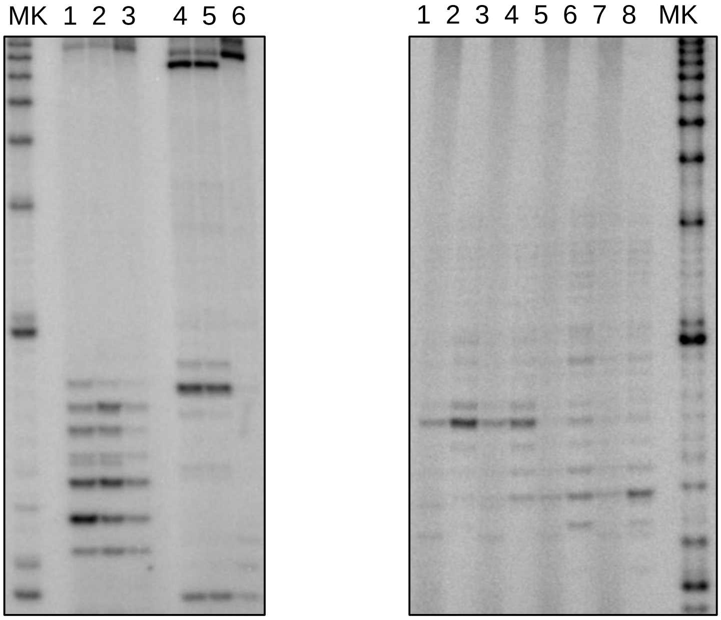

Fig. S8f

GreA	-	+	-
GreB	-	-	+
Lane	4	5	6

	St		Ni		St		Ni	
	S	P	S	P	S	P	S	P
GreB	-	-	-	-	+	+	+	+
Lane	1	2	3	4	5	6	7	8

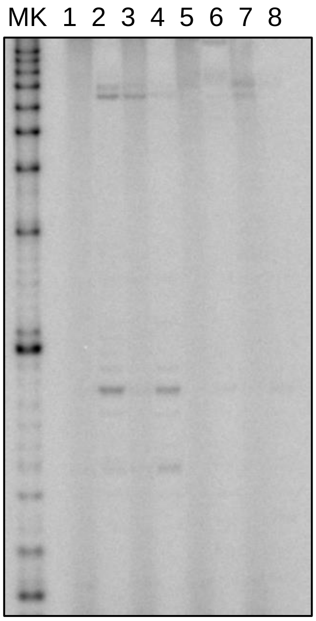

Fig. S9b

GreA - + -
GreB - - +
Lane 1 2 3

	St		Ni		St		Ni	
	S	P	S	P	S	P	S	P
GreB	-	-	-	-	+	+	+	+
Lane	1	2	3	4	5	6	7	8

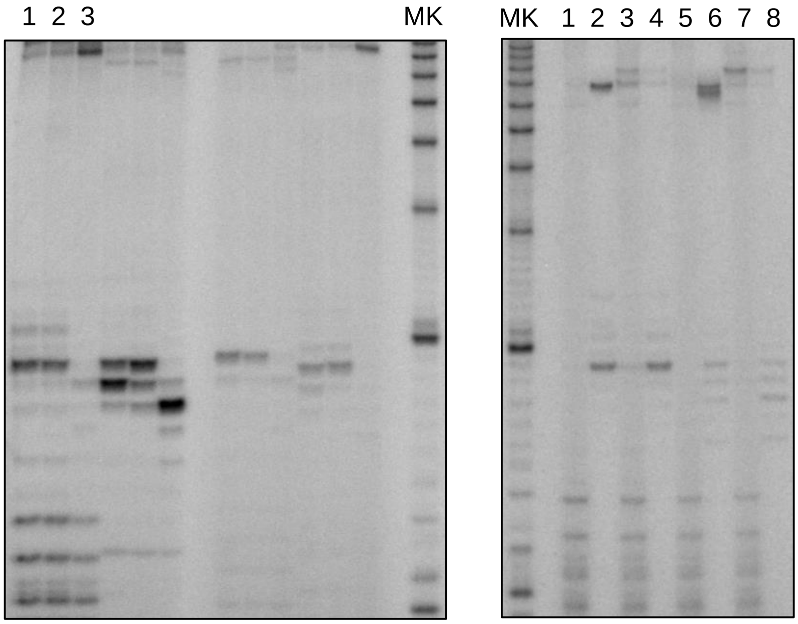

Fig. S9f

GreA - + -
GreB - - +
Lane 1 2 3
Fig. S9f (left)

GreA - + -
GreB - - +
Lane 4 5 6
Fig. S9f (middle)

GreA - + -
GreB - - +
Lane 7 8 9
Fig. S9f (right)

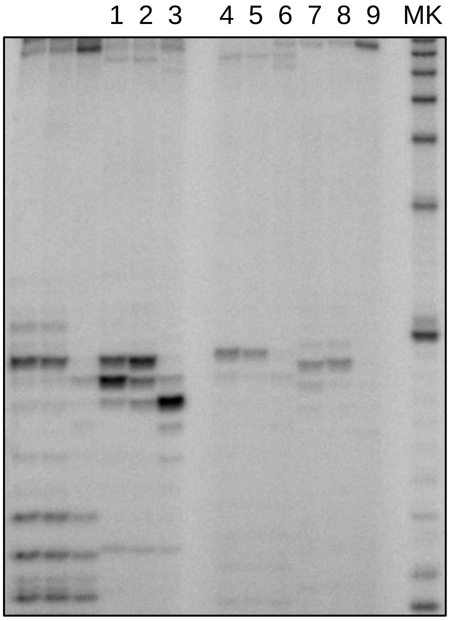

Fig. S10b Fig. S11b

WT -10R Ri- -10R Ri+
Lane 3 4 5

Fig. S10b (top, left)

WT tssR Ri- tssR R+
Lane 3 2 1

Fig. S11b (top, left)

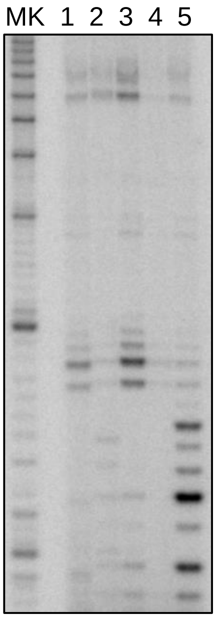

WT -10R Ri- -10R Ri+
Lane 3 2 1

Fig. S10b (top, middle)

WT tssR Ri- tssR R+
Lane 3 4 5

Fig. S11b (top, middle)

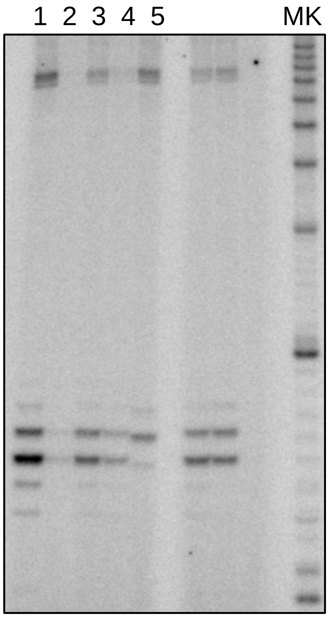

WT -10R Ri- -10R Ri+
Lane 3 4 5

Fig. S10b (top, right)

WT tssR Ri- tssR R+
Lane 3 2 1

Fig. S11b (top, right)

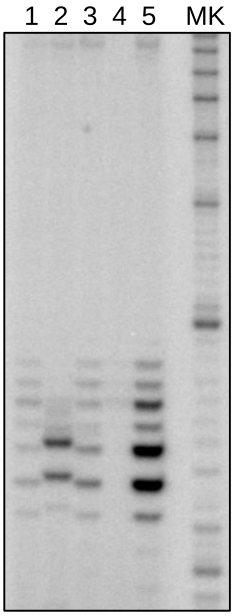

WT -10R Ri- -10R Ri+
Lane 3 2 1

Fig. S10b (bottom)

WT tssR Ri- tssR R+
Lane 3 4 5

Fig. S11b (bottom)

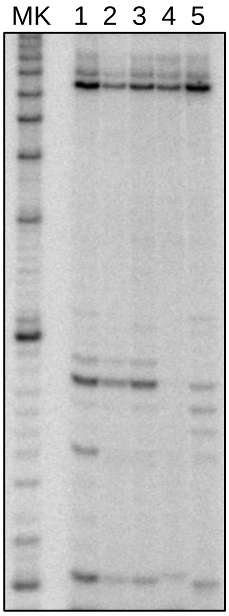

Fig. S10d Fig. S11d

WT -10R Ri- -10R Ri+
Lane 3 2 1

Fig. S10d (top, left)

WT -10LR Ri- -10LR Ri+
Lane 3 4 5

Fig. S11d (top, left)

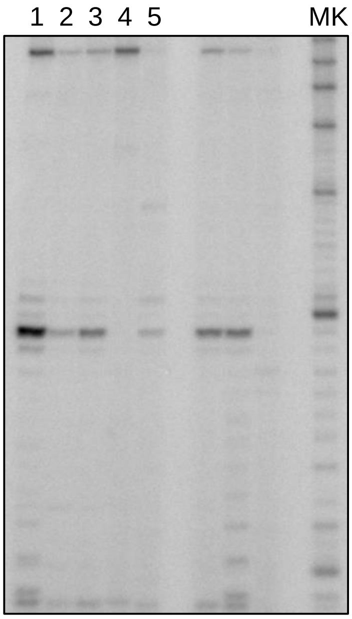

WT -10R Ri- -10R Ri+
Lane 3 2 1

Fig. S10d (top, right)

WT -10LR Ri- -10LR Ri+
Lane 3 4 5

Fig. S11d (top, right)

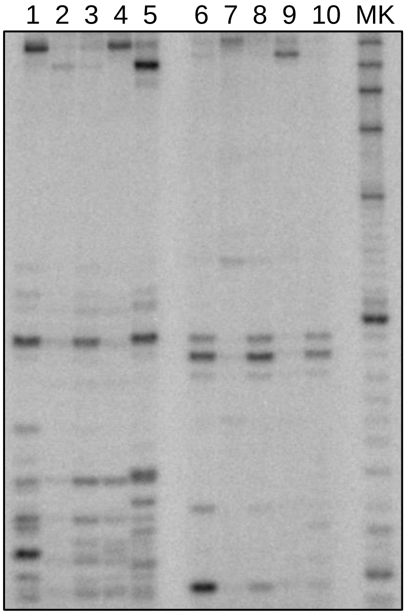

WT -10R Ri- -10R Ri+
Lane 8 7 6

Fig. S10d (bottom, left)

WT -10LR Ri- -10LR Ri+
Lane 8 9 10

Fig. S11d (bottom, left)

WT -10R Ri- -10R Ri+
Lane 3 2 1

Fig. S10d (bottom, middle)

WT -10LR Ri- -10LR Ri+
Lane 3 4 5

Fig. S11d (bottom, middle)

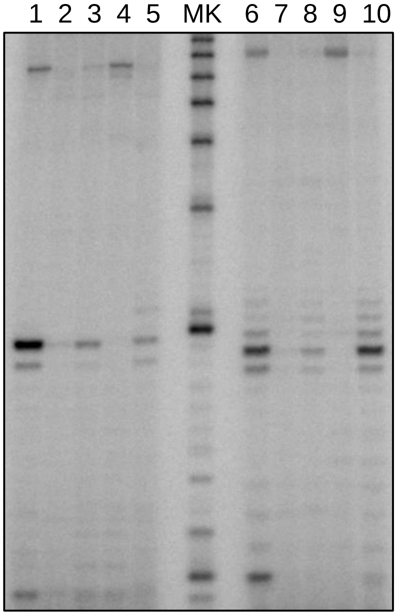

WT -10R Ri- -10R Ri+
Lane 8 7 6

Fig. S10d (bottom, right)

WT -10LR Ri- -10LR Ri+
Lane 8 9 10

Fig. S11d (bottom, right)

Fig. S12

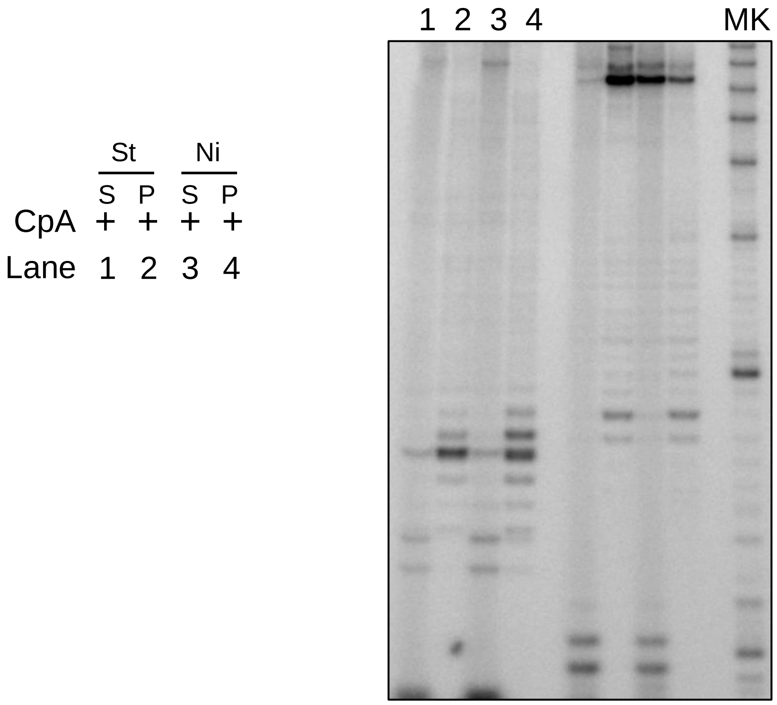

Fig. S13

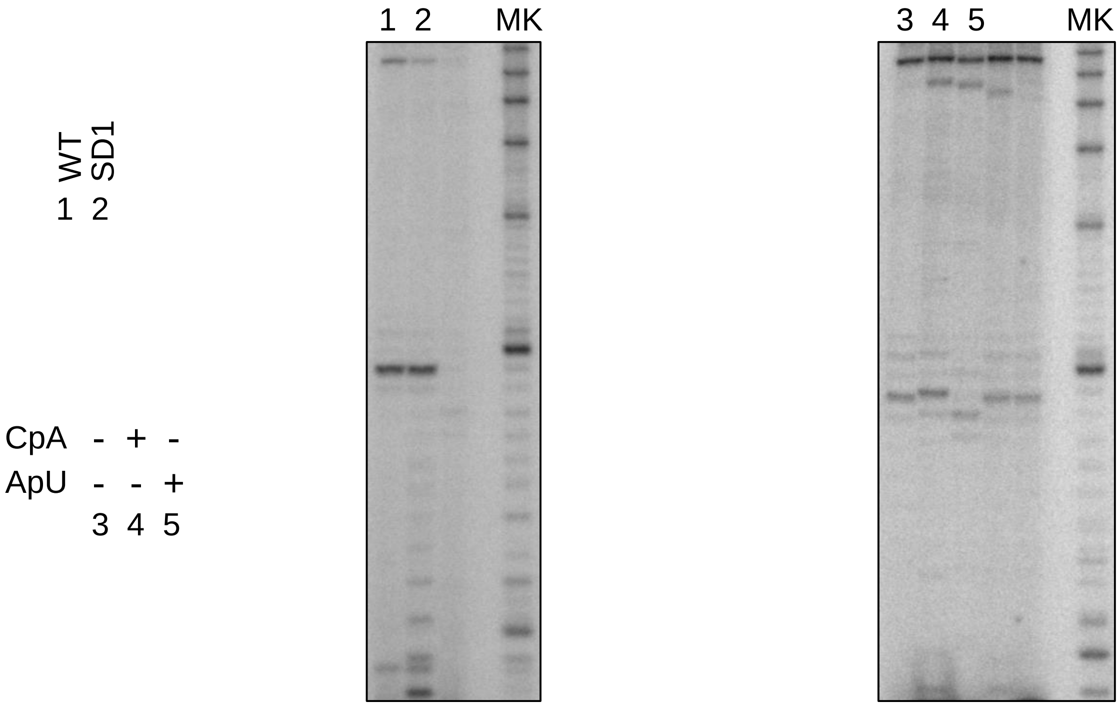

Fig. S14

	Ni	
	P	P
GreB	-	+
Lane	1	2

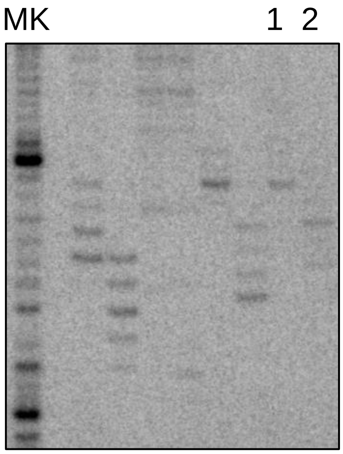

Fig. S15

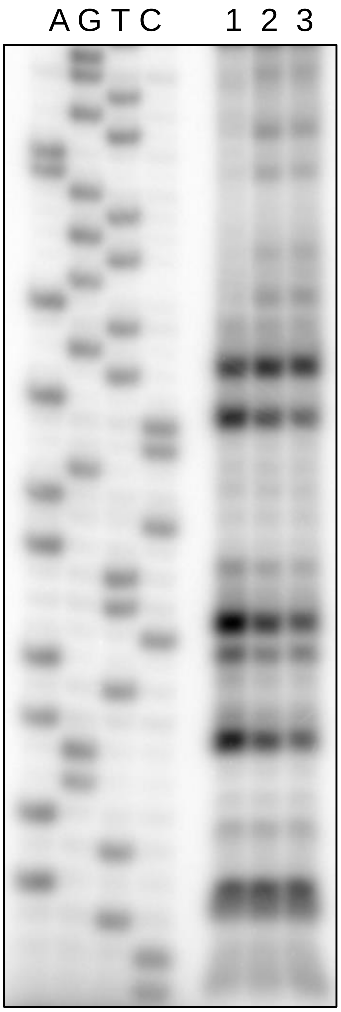

NTP	-	+	-
High NTP	-	-	+
Lane	1	2	3

Fig. S15 (left)

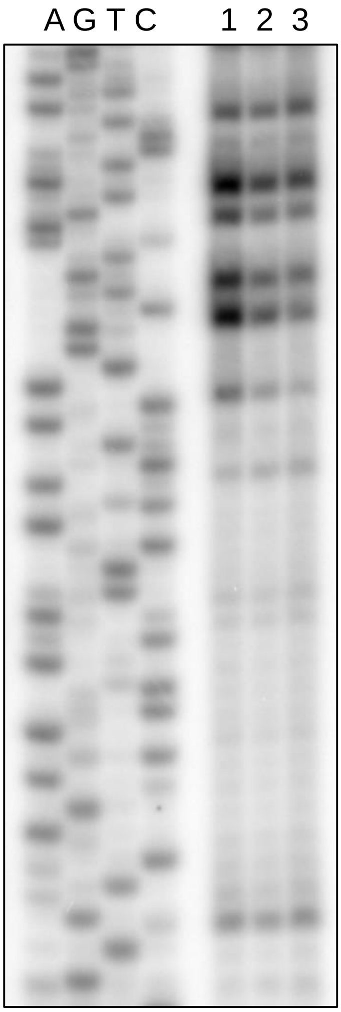

NTP	-	+	-
High NTP	-	-	+
Lane	1	2	3

Fig. S15 (right)

Fig. S18

GreB - +
Lane 1 2

Fig. S18 (left)

GreB - +
Lane 3 4

Fig. S18 (right)

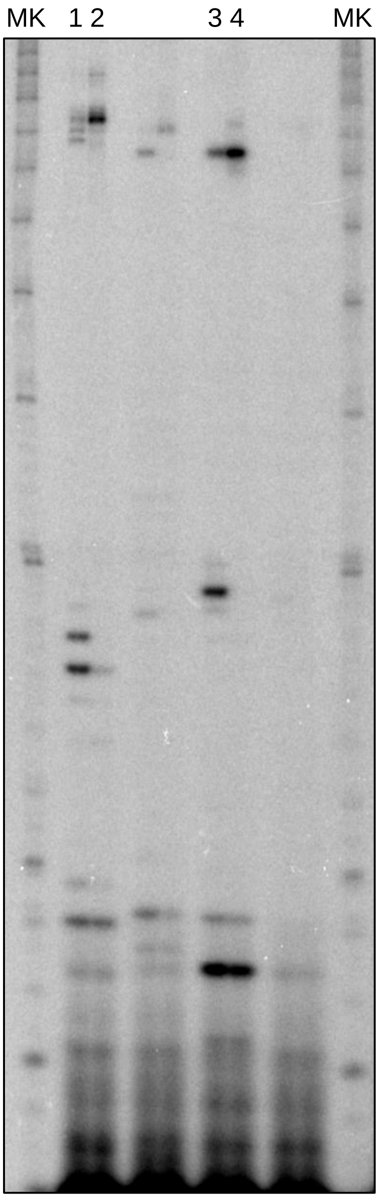

Supplement: Supplementary file 8 — Source data [file 41467_2021_21150_MOESM8_ESM.zip › Source data/Source data of uncropped gel images.pdf]
