# Supplementary material for: Nascent RNA sequencing identifies a widespread sigma70-dependent pausing regulated by Gre factors in bacteria
Source: Nat Commun. 2021 Feb 10;12:906. doi: 10.1038/s41467-021-21150-2 (PMC7876045; doi:10.1038/s41467-021-21150-2)
Supplement: Supplementary file 2 — Peer Review [file 41467_2021_21150_MOESM2_ESM.pdf]

Reviewer #1 (Remarks to the Author):

The paper presents strong evidence for the wide prevalence of sigma 70 dependent RNA polymerase pausing in E. coli, which has mostly been investigated in vitro. The authors utilise an adapted RNase I footprinting NET-seq technique to gain insight into these pauses. They reveal that the pauses fall into two categories, promoter proximal and promoter distal, based on to the length of the transcript upon pausing. The paper then provides an in-depth characterisation of both classes of pauses using the NET-seq data supported by in vitro experiments.
Throughout the paper, the authors provide evidence that the secondary channel factors GreA and GreB rescue the promoter paused complexes. The genes possessing the sigma 70 dependent pauses encode for transcriptional regulators which facilitate a response to environmental changes. The expression of GreA and GreB is also affected by the cellular environment suggesting the pauses, rescued by GreA/B, function in the response to environmental changes.

Previously, these promoter proximal pauses have only been investigated using specific examples. The ability of Gre factors to rescue the paused complexes has also previously been described. However, by using an NETseq in vivo approach supported by in vitro biochemistry, this paper provides convincing evidence that furthers our understanding of the prevalence of these pauses. Additionally, the authors provide some evidence for a biological relevance of these pauses; they function to regulate genes expressed in response to environmental perturbations. The findings of this paper would be of interest to those within the field.

I think the work is convincing and as such, additional experiments aren't really needed. However, below are further, more specific comments.

Line 112: "12211 pauses in σ70-ΔgreAB cells versus 6498 pauses in β'-ΔgreAB". I'd change that "versus" to "and".

End of Line 127 : Shouldn't it read "G1 pauses in ΔgreAB cells" instead of "β cells"

Paragraph starting on line 144: It would be nice to mention the RNAseq results. Might help changing the colours used for RNAseq tracks in Figures 3e and 3f. The ΔgreAB is mostly overlapping and so doesn't match the colour of the legend. It's not critical but could help with clarity.

Line 255: 70% of all G1 peaks identified or 70% of all RNAP peaks? Figure 6a implies its only 70% of G1 peaks from the β dataset.

Line 259/Figure 6b: Why does the figure show data from the ΔgreAB strain? Surely in this strain the expression of G1 containing genes will be down as GreA/B will not be there to rescue the paused elongation complexes?

Line 322: should read: "required to investigate the role of the robust.."

Line 323: One of the main conclusions of reference 18 (Deighan et al., 2011) is that Sigma70 dependent pauses increases the amount of sigma70 associated with the elongation complex. The authors could mention that here or elsewhere in the discussion. Furthermore, reference 18 discusses λPR proximal and distal pause sites which differ from those found in this paper. Are these pause sequences specific to the phage? The relationship between λPR and the pauses presented here could be discussed.

Figure 4f: Is it fair to say increasing Ri+ increases pause strength when there's not a significant difference?

Figure 5e: Perhaps include the blue traces in the quantification traces for GreB? From the gel, its

difficult to see that the transcription bubble is reduced in size

Figure 6e/Line 272: Would it be possible from the ΔgreAB mutant RNAseq data to show that the genes regulated by the proteins encoded by the sigma70 dependant pause controlled genes have reduced expression? This would strengthen the argument that these pauses control gene expression in response to environmental changes.

Extended data Figure 8b: In solution, GreB cleaves the transcript to 14 nt. However, the immobilised complexes are cleaved to down to 13 and 12 nt. Is this difference due to how the complexes are radiolabelled? If not, what's causing this difference?

Extended data Figure 17: The labels on the graph are not clear. Specifically, what is 0.5 referring to? OD600?

Reviewer #2 (Remarks to the Author):

This is a subject worthy of investigation, and the paper reports a substantial accomplishment. Previous reports of sigma70-dependent pausing involve a small number of sites, with little or no information about their function in bacterial (as opposed to bacteriophage) gene expression. This paper performs a global analysis of pausing in E. coli and apparently identifies a large number of novel sigma70-dependent pauses. That said, there are many confusing aspects to the paper.

A major problem with the analysis is the failure to relate these findings to the well-studied and characterized process of abortive initiation, and to previous studies that characterized the process of initial transcription and RNAP escape. Such studies have identified a rate-limiting step with a scrunched initial transcribing complex in which RNAP holoenzyme remains bound to the promoter, from which transcription is known to abort (in vitro and in vivo) and have clearly related the nature of this phenomenon to the strength of the promoter. This is no doubt the origin of what the authors describe as pauses from their set of G1p promoters. No one would designate this process as sigma70-dependent pausing, which is universally used to describe the pauses in the authors' set of G1d promoters. Certainly, the step that give rises to abortive transcripts may be an important site of regulation, as many previous workers have proposed.

The reviewer simply cannot understand Figure 1b, which appears to illustrate the central isolation scheme of the paper. The transcripts isolated by sigma70 affinity should be a subset of those isolated by beta prime affinity, because sigma70 is only present when bound to core enzyme, but that is not what the figure appears to show. How is Figure 1b related to Figure 1d?

Lines 43-45: a function of the pausing site in coordinating RNAP movement with concurrent translation was conjectured but certainly never shown.

Line 83: Where is it shown that the complexes are capable of elongation?

Line 104 mentions 7412 and 3543 pauses. Really? Is there even this number of promoters?
The sentence further says these numbers apply to "sigma70-WT" and "beta prime-WT" cells. Is this supposed to mean "identified by sigma70 affinity selection" and "identified by beta prime affinity selection"?

The discussion of sigma "hopping" gives rise to the impression that sigma alone might hop from promoter to pause-inducing site. But it certainly only hops in the context of the holoenzyme. Then, it is not clear what the importance of sigma binding sites on the same face of the DNA then would be.

The reviewer has no expertise in the statistical analysis involved in the global sequencing studies.

Reviewer #3 (Remarks to the Author):

Sun et al present an elegant study of genome-wide sigma70-dependent RNAp pausing. The authors purify nascent active elongation complexes and sequence RNA protected by these complexes from RNAse I digestion wherein the 3'-end positions of these RNAs indicate the precise sites of pausing. Sequencing is validated by classic biochemistry, which is appreciated. This also allowed the authors to propose the mechanisms of this pausing that involve promoter-like interactions of the elongating holoenzyme with -10-like elements and that these pausing events are GreA/B-controlled. A new finding is that this pausing is quite common across the genome and that it may involve multiple mechanisms that have been dissected by careful analysis of protected RNA 3'-ends and GreA/B contributions.

Technical points.

- Figure 1b. The relevant text (lines 84-86) implies that all RNAP complexes thus purified are active. The labeling experiment shows that at least some complexes are active, but I do not think it proves that all complexes are active. Double check the wording?

- Figure 1c. What is the point of showing Pearson correlation in a main figure because extended fig 1 shows correlation just fine. Was showing Pearson instead of rank-orders intended as validation of normalization between replicates done in some other way? There appears to be no other reason to show this. If so, I could not find information on how individual replicates were normalized. I apologize in advance if I had missed it.

- What is the maximum possible size of RNA protected by the RNA polymerase under complete RNAse digestion conditions? It seems to me that the longer, 20+nt reads observed in abundance in GreA/B deletant libraries indicate that RNAse digestion is far from complete. There is clearly a difference between WT and GreA/B datasets, but I am not certain whether these values can be taken at face value.

- Lines 91-92. The reads might be too short to map to the genome indeed. However, if a majority of reads from sigma libraries come from TSSs, could authors map these discarded reads from these datasets to known TSS regions instead of the entire genome? Even though the mapping is expected to be less certain than that to the genome, these reads may end up carrying information.

- Re: shorter (12-17nt) RNAs in GreAB-minus libraries (lines 98-99). I do not understand why shorter rather than longer RNAs are observed in the absence of GreA/B. The ms by Marr and Roberts cited here does show shortening of RNAs with GreA/B addition as expected. What is going on here – a competition between sigma70 and GreA/B? Is there anything known about whether GreA/B can actually suppress pausing or is this the finding being made here? This is also talked about in the next section, but there is no indication as to whether this is new or not. The same point is being made in several places throughout, but never decisively. As a side note, it is possible that mapping the aforementioned short reads to TSSs – or proving that they do not map to known TSSs – may help address this.

- Figure 2C. Enrichment should be also shown normalized against representation of each feature in the genome. Also, what is the significance of showing pauses mapping to coding versus noncoding (presumably 5'-UTR) portions of genes?

- Based on Figures 2D and 2E, G0 pauses seem to be dominating the wild type sigma70 libraries. This prominence makes them hard to ignore outright. If these are generated by initiation from upstream

promoters as suggested in the text (what is meant here by upstream promoter?), why are these transcripts selectively much more sensitive to GreA/B? Why this phenomemon seems to be exclusive to sigma libraries also makes no sense to me.

• Comparison of different dinucleotide primers to distinguish contribution of DNA versus RNA is much appreciated. Is there a reason why the Runoff transcript in 4G lane 5 is lower and not higher in intensity than in lane 4 as would be expected if this were due to lower pausing and higher readthrough efficiency?

• Figures 5D and E are extremely hard to understand as presented. First of all, no background DNA reactivity appears to be shown, which would be a serious omission of a control. Secondly, the footprints may be "zoomed in" too much so it is unclear where the bubble begins or ends. Extended figure 15 shows the correct region much more clearly. Is T7A1 a pauseless promoter? If so, it belongs in the main panel to show a contrast with T1 and T3 NTP-dependent reactivity in main 5D.

• Figure 5E example of paused promoter is particularly hard to understand. Perhaps insert a small arrow to where the difference +/- is? Secondly, the point mutation makes me even more confused. What the mutation may have caused is to create a new promoter there that forms a very nice bubble that is not dependent on NTPs. Given that, I do not know if petering out of green line signal in downstream regions is even real – I do not see it visually on the gel itself.

• Is there a correlation between the counts of short RNAs at promoters and expression of the same gene? The latter can be probably inferred from beta' datasets in gene bodies if other data are not available. In other words, is pausing repressive or part of a normal transcription cycle?

• Figure 1 legend makes speculations about the origin of <16nt RNAs. I wonder if this material should be in Discussion rather than figure legends, but this is of course up to the authors. In followup future experiments these may be possible to tell apart by ligating RNA pools +/- alkaline phosphatase pre-treatment.

Reviewer 1 (Remarks to the Author)
The paper presents strong evidence for the wide prevalence of sigma70 dependent RNA polymerase pausing in E. coli, which has mostly been investigated in vitro. The authors utilise an adapted RNase I footprinting NET-seq technique to gain insight into these pauses. They reveal that the pauses fall into two categories, promoter proximal and promoter distal, based on to the length of the transcript upon pausing. The paper then provides an in-depth characterisation of both classes of pauses using the NET-seq data supported by in vitro experiments.

Throughout the paper, the authors provide evidence that the secondary channel factors GreA and GreB rescue the promoter paused complexes. The genes possessing the sigma70 dependent pauses encode for transcriptional regulators which facilitate a response to environmental changes. The expression of GreA and GreB is also affected by the cellular environment suggesting the pauses, rescued by GreA/B, function in the response to environmental changes.

Previously, these promoter proximal pauses have only been investigated using specific examples. The ability of Gre factors to rescue the paused complexes has also previously been described. However, by using an NET-seq in vivo approach supported by in vitro biochemistry, this paper provides convincing evidence that furthers our understanding of the prevalence of these pauses. Additionally, the authors provide some evidence for a biological relevance of these pauses; they function to regulate genes expressed in response to environmental perturbations. The findings of this paper would be of interest to those within the field.

I think the work is convincing and as such, additional experiments aren't really needed. However, below are further, more specific comments.

Line 112: "12211 pauses in σ70-ΔgreAB cells versus 6498 pauses in β'-ΔgreAB". I'd change that "versus" to "and".
Response: The sentence was modified (line 111).

End of Line 127 : Shouldn't it read "G1 pauses in ΔgreAB cells" instead of "β' cells"
Response: We highly appreciate the reviewer's careful reading of this part. The edited sentence now states that "Most G0 and G2 pauses were substantially weaker than the G1 pauses in both, WT and Δ*greAB* cells (Supplementary Table 1)" (line 126-127). We also added the reference to Supplementary Table 1.

Paragraph starting on line 144: It would be nice to mention the RNAseq results. Might help changing the colours used for RNAseq tracks in Figures 3e and 3f. The ΔgreAB is mostly overlapping and so doesn't match the colour of the legend. It's not critical but could help with clarity.
Response: We agree with the reviewer's suggestion. RNA-seq confirmed the 1.3- and 2.5-fold higher number of reads in a 200 bp region immediately downstream of the *mraZ* and *yieE* pause sites in WT compared with the Δ*greAB* cells (Fig. 3e, f, bottom). We have added this sentence summarizing the RNA-seq results to the text (line 154-156).

We adjusted the color of RNA-seq tracks (Fig. 3e, f) as the reviewer recommended.

Line 255: 70% of all G1 peaks identified or 70% of all RNAP peaks? Figure 6a implies its only 70% of G1 peaks from the β dataset.
Response: That's correct, the wording should be "~70% of all G1 peaks from RNAP". We have edited the sentence (line 263).

Line 259/Figure 6b: Why does the figure show data from the ΔgreAB strain? Surely in this strain the expression of G1 containing genes will be down as GreA/B will not be there to rescue the paused elongation complexes?
Response: There are several reasons for presenting the Δ*greAB*, but not WT data in this figure. First, as the reviewer pointed out, the genes harboring G1 pauses had higher transcription level in WT compared with Δ*greAB* cells as shown in Fig. 6d. It means that even if these genes exhibited significant difference compared to the control genes lacking G1 pauses in WT cells, this may be still not true for Δ*greAB* cells, because of the less prominent effect. Second, G1p and G1d pauses from Δ*greAB* cells were analyzed in Fig. 6a. Therefore, we felt more logical to apply the TPM data from Δ*greAB* strain to the graph of Fig. 6b. This also placed these data to the same genetic context.

Line 322: should read: "required to investigate the role of the robust.."
Response: The sentence was modified as suggested (line 353).

Line 323: One of the main conclusions of reference 18 (Deighan et al., 2011) is that Sigma70 dependent pauses increases the amount of sigma70 associated with the elongation complex. The authors could mention that here or elsewhere in the discussion. Furthermore, reference 18 discusses λPR proximal and distal pause sites which differ from those found in this paper. Are these pause sequences specific to the phage? The relationship between λPR and the pauses presented here could be discussed.
Response: Based on the conclusion made in ref. 18, the G1 pausing could increase retention of σ^{70} in the elongation complexes at a large distance from the original promoter to additionally reinforce pausing caused by σ^{70}-mediated recognition of the -10-like sequences. We have added this thought to Discussion (lines 350-352).

The sequence and the distance between the λpR' promoter elements are similar to those of the bacterial promoters coding for G1d pauses. λpR' has a canonical -10 region, a 6-nt spacer between -10 region and TSS and a -10-like region. The only difference is that the proximal -10-like region of the λpR' promoter shifts 1 bp toward TSS compared to G1d promoters. The G2 pauses that we elected not to analyze here also have a AT-rich region 13-21 bps upstream the pause sites (as shown in the figure below), which matches the location of the distal -10-like region of λpR' promoter.

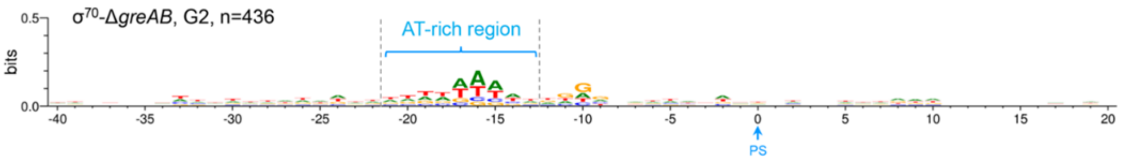

The distances from TSS to the promoter-proximal (16-17 nt) and the distal (35-37 nt) pause sites at λpR' promoter also match those for G1d (16-20 nt) and G2 (31-39 nt) pauses. These similarities strongly indicate that the σ^{70}-dependent *in vitro* pauses at λpR' promoter are not specific to the phage and have the same mechanism as the σ^{70}-dependent G1d and G2 pauses. We added this information to Discussion (lines 310-314).

Figure 4f: Is it fair to say increasing Ri+ increases pause strength when there's not a significant difference?

Response: In our analysis, we meant to say that the mutations that decreased Ri of tssR significantly reduced G1p pausing. The mutations increasing Ri of tssR carrying the sub-optimal tssR sequence moderately increased the pause strength of a subset of the G1p pauses. To address the reviewer's concern, we broke the long sentence into two parts to make it more clear (lines 195-199).

Figure 5e: Perhaps include the blue traces in the quantification traces for GreB? From the gel, its difficult to see that the transcription bubble is reduced in size

Response: We agree with the reviewer and have added the quantification profile of the blue lane to this figure (Fig. 5e).

Figure 6e/Line 272: Would it be possible from the ΔgreAB mutant RNAseq data to show that the genes regulated by the proteins encoded by the sigma70 dependent pause controlled genes have reduced expression? This would strengthen the argument that these pauses control gene expression in response to environmental changes.

Response: To follow the reviewer's suggestion, we analyzed expression of the regulon genes regulated by the G1-containing genes coding for transcription regulators in WT and Δ*greAB* cells. We found that transcription of the regulon genes regulated by G1 repressors (such as TrpR; see the graph shown below), was significantly increased in Δ*greAB* cells, whereas transcription of some regulon genes of G1 activators (such as GntR) was reduced, which was consistent with our model presented in Fig. 6e. However, similar analysis was hardly feasible for many other regulators since the same factor could function as either repressor or activator depending on the position of a factor-binding site relative to core promoter elements. We also noted that transcription of many groups of the G1 regulators regulon genes was not affected by Gre deletion suggesting that, in addition to the regulation by Gre factors, other sophisticated regulatory networks were present in *E. coli* cells.

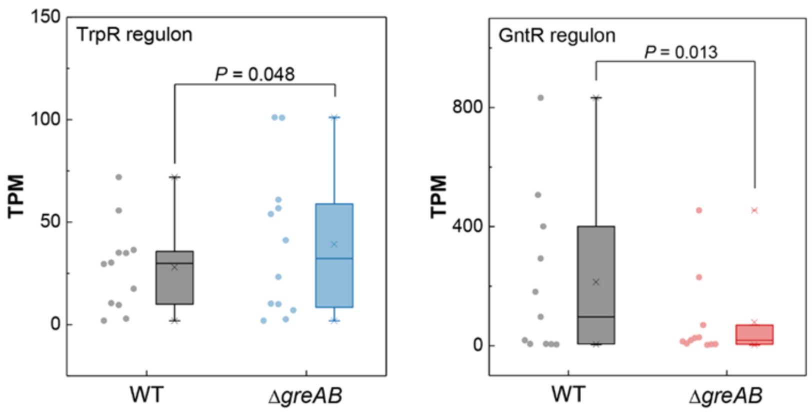

Extended data Figure 8b: In solution, GreB cleaves the transcript to 14 nt. However, the immobilised complexes are cleaved to down to 13 and 12 nt. Is this difference due to how the complexes are radiolabelled? If not, what's causing this difference?

Response: We thank the reviewer for bringing our attention to the different patterns of GreB-induced cleavage. The transcripts made in solution (Supplementary Fig. 8b, left) and on beads (Supplementary Fig. 8b, right) were similarly labeled at the 5' ends by employing [γ-^{32}P] ATP to initiate transcription. We attributed this difference to a substantially higher local concentration of RNAP on beads as opposed to RNAP in solution. This high local concentration might facilitate binding of GreB to RNAP immobilized on beads by promoting GreB exchange between molecules of RNAP residing close to each other on the bead surface. This effect may explain the more efficient cleavage of the nascent RNA in the solid phase observed in Supplementary Fig. 8b.

Extended data Figure 17: The labels on the graph are not clear. Specifically, what is 0.5 referring to? OD600?

Response: Yes, it should be OD_{600}. As the reviewer pointed out, we have modified Supplementary Fig. 17 and added the more detailed information to its legend (Supplementary Figures, lines 140-142).

Reviewer 2 (Remarks to the Author):

This is a subject worthy of investigation, and the paper reports a substantial accomplishment. Previous reports of sigma70-dependent pausing involve a small number of sites, with little or no information about their function in bacterial (as opposed to bacteriophage) gene expression. This paper performs a global analysis of pausing in E. coli and apparently identifies a large number of novel sigma70-dependent pauses. That said, there are many confusing aspects to the paper.

A major problem with the analysis is the failure to relate these findings to the well-studied and characterized process of abortive initiation, and to previous studies that characterized the process of initial transcription and RNAP escape. Such studies have identified a rate-limiting

step with a scrunched initial transcribing complex in which RNAP holoenzyme remains bound to the promoter, from which transcription is known to abort (in vitro and in vivo) and have clearly related the nature of this phenomenon to the strength of the promoter. This is no doubt the origin of what the authors describe as pauses from their set of G1p promoters. No one would designate this process as sigma70-dependent pausing, which is universally used to describe the pauses in the authors' set of G1d promoters. Certainly, the step that give rises to abortive transcripts may be an important site of regulation, as many previous workers have proposed.

Response: We agree with the reviewer that the abortive transcription should be discussed in a context of G1 pauses described in our work. The abortive initiation is one of the best studied regulatory process characterized *in vitro* at a large number of bacterial promoters and also demonstrated for RNA polymerase II. However, a correlation between the robust abortive synthesis and the strong promoter-proximal pausing (G1p and G1d) and a role of DNA scrunching as a driving force for both processes remain obscured. One of the advantages of the RNET-seq method is its ability to purify elongation complexes in their native state without using highly biased DNA/RNA/protein crosslinking. However, the RNAP complexes with DNA and RNA need to be sufficiently stable to sustain multiple purification steps including treatment with high salt. This limits the ability of RNET-seq in tackling the transient and structurally unstable abortive complexes unless the short abortive 2-10-nt RNAs form stable complexes with RNAP. Even if the stable retention of the abortive products occasionally occurs in RNET-seq libraries, these short RNAs cannot be uniquely mapped to the genome (the uniquely mapping cutoff for *E. coli* genome is about 12-14-nt). By acknowledging this limitation, we could only guess that the G1p and G1d pauses carrying the short RNAs derived from the early transcription elongation complexes as opposed to the RNA-stabilized abortive complexes. Nevertheless, we seriously considered the reviewer's comment that the G1 pauses and the robust abortive synthesis might both result from the strong binding of σ^{70} to the corresponding promoters. This possibility certainly warranted an experimental testing taking into account that Gre proteins have been shown to affect the yield and size of the short products in vitro (Lilian M. H. et al. PNAS. 1995).

To address the reviewer's comment on the abortive transcription, we repeated the single round in vitro transcription experiment using the G1p (*mraZ*) and G1d (*yieE*) promoters described in Fig. 3g, h. To identify the short aborted RNAs, we analyzed these samples using long high percentage sequencing gel to achieve separation the short abortive and the longer >12-nt G1 RNA species. As we expected, GreB eliminated the >12-nt G1 RNAs by promoting their extension to the runoff products. In contrast, the shorter abortive RNAs appeared to be GreB resistant and their yield was barely affected by GreB at both G1 promoters (the data shown below). In combination with the RNET-seq data (Fig. 3e, f), this result made us convinced that the G1p and G1d pauses primarily derived from σ^{70}-dependent early elongation step as opposed to the pathway involving retention of the short abortive products in RNAP. This result did not completely eliminate the possibility that the G1-like pauses in the +2/+12 abortive register might occur at some bacterial promoters. This question warrants further testing with a larger number of G1 promoters. We included a statement about the origin of G1p pauses to Discussion (lines 298-306). In general, we agree with the reviewer that the step related to

abortive transcripts is important for transcription regulation, but also admitted that our RNET-seq approach could not unambiguously answer this question in vivo.

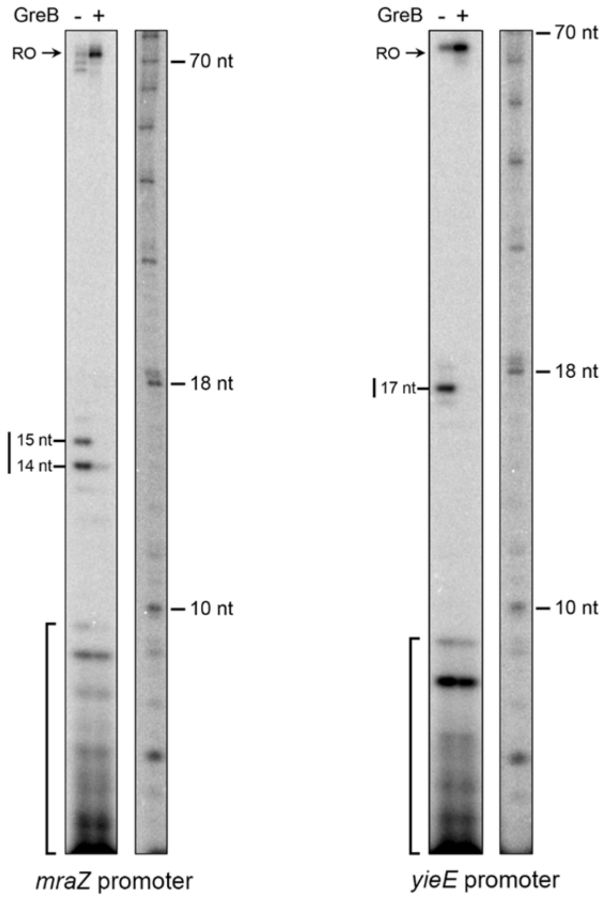

The reviewer simply cannot understand Figure 1b, which appears to illustrate the central isolation scheme of the paper. The transcripts isolated by sigma70 affinity should be a subset of those isolated by beta prime affinity, because sigma70 is only present when bound to core enzyme, but that is not what the figure appears to show. How is Figure 1b related to Figure 1d?
Response: We apologize for the confusion. In fact, Fig. 1a illustrated the central isolation scheme of RNET-seq. In vitro incorporation of [α-^{32}P] UTP and the RNA cleavage stimulated by GreB as shown in the scheme of Fig. 1b were used to test whether the purified TECs remained intact and capable of elongation. This was a matter of our special concern because DNA/RNA/protein cross-linking was not employed to stabilize the ternary complexes during the RNET-seq library preparations.

We totally agree with the reviewer's comment that the transcripts isolated by σ^{70} affinity should represent a subset of those isolated by β' affinity. Obtaining the similar yield of the nascent RNA was necessary to construct the σ^{70} and β' RNET-seq libraries with the comparable sequencing depth. As a result, a substantially larger amount of biomass was used for constructing the σ^{70}-WT/σ^{70}-Δ*greAB* libraries compared to their β'-WT/β'-Δ*greAB* counterparts. This difference resulted in a notably different appearance in the gel of the nascent RNA

extracted from σ^{70}-WT and β'-WT cells (Fig. 1b). That's the reason why the σ^{70}-WT prep had higher amount of high (≥ 25-nt)and low (≤ 9-nt) molecular weight RNAs than the β'-WT prep. We added to the figure legend a description clarifying this technical point (lines 694-695).

We also want to emphasize that length distribution of the RNA products shown in Fig. 1b cannot be directly compared to the corresponding distribution of the nascent RNAs shown in Fig. 1c (Fig. 1d before revision). The nascent RNA species shown in Fig. 1b were labeled at the 3' ends with low concentration of [α-^{32}P] UTP, which revealed only the catalytically active TECs. In contrast, the RNA species of Fig. 1c represented ALL nascent RNAs associated with RNAP including the complexes having low or no catalytic activity. Moreover, some short RNAs that were successfully labeled in the experiment of Fig. 1b could not be uniquely mapped to the genome due to their short length, which were excluded from the analysis of Fig. 1c.

Lines 43-45: a function of the pausing site in coordinating RNAP movement with concurrent translation was conjectured but certainly never shown.
Response: We agree with the reviewer that there is no direct evidence showing that this coordination occurs in vivo. However, a significant enrichment of the consensus pausing sequences at translation start sites (ref. 6) suggests that the coupling between transcription and concurrent translation may take place in vivo (ref. 4). In addition, the cooperation between translating ribosomes and elongating RNAP was also reported previously (Sergey P. et al. Science. 2010; Manlu Z. et al. Nat Microbiol, 2019). We softened the statement as the reviewer suggested (line 37).

Line 83: Where is it shown that the complexes are capable of elongation?
Response: The result of the in vitro [α-^{32}P] UTP incorporation experiment (Fig. 1b) proves that, at least a fraction of the immobilized complexes retained the catalytic activity. Most importantly, this experiment detected only a subset of the TECs that had high catalytic activity enabling the TECs to incorporate [α-^{32}P]UTP at low concentration. To complement the labeling approach, the efficient label-free extension of the nascent RNA in TECs pulled down on Ni-NTA beads (ref 7, Fig. S1B as shown below) further demonstrated that a vast majority of the elongation complexes was active. Note that the label-free experiment involved high concentration of all 4 NTPs leading to the better recovery of the TECs having low catalytic activity.

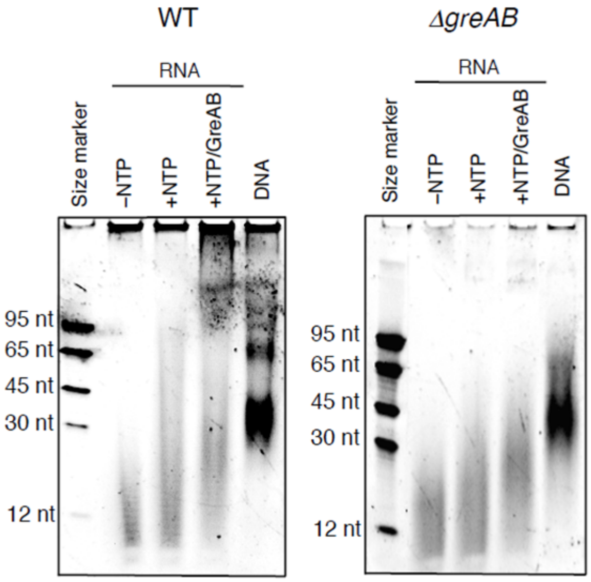

Line 104 mentions 7412 and 3543 pauses. Really? Is there even this number of promoters? The sentence further says these numbers apply to "sigma70-WT" and "beta prime-WT" cells. Is this supposed to mean "identified by sigma70 affinity selection" and "identified by beta prime affinity selection"?

Response: Basically, these are the total number of pauses identified by RNET-seq, which are not restrict to promoter-proximal G1 pauses. Several reasons indicate that these numbers are relative reasonable compared with the total number of reported TSSs. 1) We found that a surprisingly large number of G1 pauses that we identified were consistent with the recent published TSS mapping efforts that use different mapping approaches. Although only ~5200 TSSs are reported in the RegulonDB database, the dRNA-seq (Maureen K.T. et al. J Bacteriol. 2015) and the Cappable-seq (Laurence E. et al. BMC Genomics. 2016) identify a substantially larger numbers of TSSs in *E. coli* grown in rich and minimal media (~8500 and ~16500 sites, respectively). 2) Although many pause sites identified by our RNET-seq were located near annotated promoters, a substantial fraction of these sites was localized at a significant distance from the annotated TSS further increasing the total number of the pauses reported in our work. 3) Moreover, some promoter-proximal regions identified by RNET-seq contained more than one pause sites.

According to the reviewer's suggestion, we included the explanation of "σ^{70}-WT" and "β'-WT" in the mentioned sentence (lines 103-104).

The discussion of sigma "hopping" gives rise to the impression that sigma alone might hop from promoter to pause-inducing site. But it certainly only hops in the context of the holoenzyme. Then, it is not clear what the importance of sigma binding sites on the same face of the DNA then would be.

Response: We concur with the reviewer's argument. The -10 element and the -10-like sequence located on the same face of the DNA helix may facilitate hopping (or repositioning on DNA) of

the σ_2 domain as opposed to re-positioning the entire σ^{70} of RNAP. Although the latter scenario is unlikely to happen at G1 pauses, it cannot be completely excluded. The domain hopping/switching model is supported by the published data showing the similar contacts with DNA in RPo and G1d-paused TEC in the structure of arrested complex at λpR' promoter (Jing S. et al. Nat Commun. 2019). The dsDNA constrained by the σ_2 domain may help maintain the interaction between σ_4 and the promoter -35 element to further stabilize the paused elongation complex. Based on the reviewer's suggestion, we modified the corresponding part of Discussion accordingly (lines 316, 319-323).

The reviewer has no expertise in the statistical analysis involved in the global sequencing studies.
Response: The manuscript is co-authored by the two senior members of the NCI Bioinformatics Core Facility (Peter FitzGerald and Carl McIntosh), who have high expertise in bioinformatic analysis of NGC data including RNA-seq and NET-seq.

Reviewer #3 (Remarks to the Author):

Sun et al present an elegant study of genome-wide sigma70-dependent RNAp pausing. The authors purify nascent active elongation complexes and sequence RNA protected by these complexes from RNAse I digestion wherein the 3'-end positions of these RNAs indicate the precise sites of pausing. Sequencing is validated by classic biochemistry, which is appreciated. This also allowed the authors to propose the mechanisms of this pausing that involve promoter-like interactions of the elongating holoenzyme with -10-like elements and that these pausing events are GreA/B-controlled. A new finding is that this pausing is quite common across the genome and that it may involve multiple mechanisms that have been dissected by careful analysis of protected RNA 3'-ends and GreA/B contributions.

Technical points.
Figure 1b. The relevant text (lines 84-86) implies that all RNAP complexes thus purified are active. The labeling experiment shows that at least some complexes are active, but I do not think it proves that all complexes are active. Double check the wording?
Response: We agree with the reviewer that Fig. 1b depicted only those TECs that were capable for incorporation of [α-^{32}P] UTP at low concentration. The similar comment was made by Reviewer 2. However, our statement that the majority of the immobilized TECs were active was also supported by an additional experiment showing the efficient LABEL-FREE extension of the nascent RNA in TECs pulled down on Ni-NTA beads (ref. 7, Fig. S1B as shown below). Note that, in contrast to the data of Fig. 1b, the experiment of Fig. S1B (ref. 7) visualized ALL nascent RNAs. Also, the NTP chase reaction involved high concentration of all 4 NTPs further promoting the RNA elongation by the TECs having low catalytic activity. Based on the reviewer's comment, we modified this part to avoid confusion (line 79).

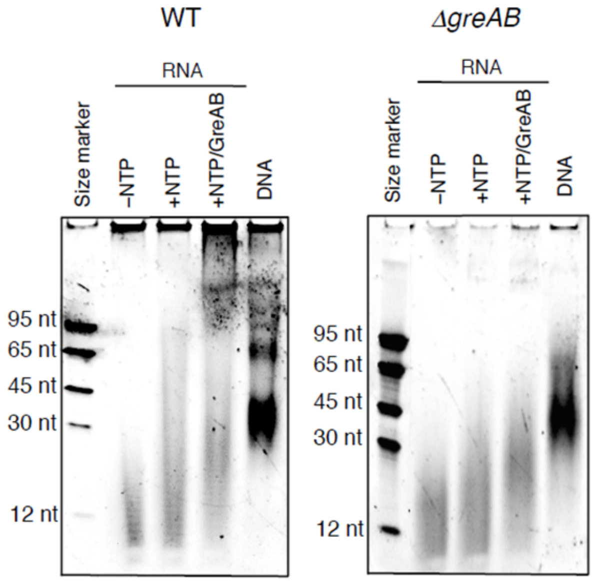

Figure 1c. What is the point of showing Pearson correlation in a main figure because extended fig 1 shows correlation just fine. Was showing Pearson instead of rank-orders intended as validation of normalization between replicates done in some other way? There appears to be no other reason to show this. If so, I could not find information on how individual replicates were normalized. I apologize in advance if I had missed it.

Response: Originally, Fig. 1c was used to summarize the correlation between two biological replicates as shown in Supplementary Fig. 1. As the reviewer suggested, we removed Fig. 1c.

What is the maximum possible size of RNA protected by the RNA polymerase under complete RNAse digestion conditions? It seems to me that the longer, 20+nt reads observed in abundance in GreA/B deletant libraries indicate that RNAse digestion is far from complete. There is clearly a difference between WT and GreA/B datasets, but I am not certain whether these values can be taken at face value.

Response: Based on our biochemical analysis, RNAP core enzyme occupying the pre-translocated register protects 18-nt RNA from RNase I digestion. Backtracking extends this protection to the upstream direction. This happens because during backtracking the 3' RNA end remains protected in the secondary pore of RNAP while the upstream RNA is poured in the ssRNA-binding channel making it inaccessible to RNase I. Although we didn't test it in vitro, our RNET-seq results indicated that the RNA 3' end remained protected from RNase I digestion after backtracking at up to 10-bp distance, which was likely to be responsible for generation of the 20+ nt reads including the longest 28-nt (18+10) reads. These long RNET-seq reads were detected in vivo, particularly in the cells lacking Gre factors.

When isolating the elongation complexes, RNase I digestion was performed 3 times during cell lysis and, then before and after release of RNAP from the nucleoid after treatment with DNase I. To ensure that the digestion was complete, we carefully titrated RNase I and used it at a high concentration as indicated in the Methods section (lines 382-383, 383-385, 391-394). The resulting RNET-seq data showed a relatively narrow and highly reproducible distribution of the

read length at each pause site and also at other locations throughout *E. coli* genome. Most importantly, this distribution was consistent with the in vitro data on the individual TECs treated with high doses of RNase I. If the digestion of TECs in the lysate was not complete as the reviewer argued, this would produce a broad range of the read length. We did not observe these broad peaks in the RNET-seq data. Note that the same RNET-seq condition (including the RNase I treatment) was also applied to RNET-seq in *B. subtilis* where 20+ nucleotide reads were rarely observed (Yakhnin A. V. et al. PNAS. 2020). We attributed this difference to a very poor backtracking capability of *B. subtilis* RNAP compared to the *E. coli* enzyme. Collectively, these evidences indicate that the RNase I digestion was complete in our conditions.

Lines 91-92. The reads might be too short to map to the genome indeed. However, if a majority of reads from sigma libraries come from TSSs, could authors map these discarded reads from these datasets to known TSS regions instead of the entire genome? Even though the mapping is expected to be less certain than that to the genome, these reads may end up carrying information.
Response: This is a good point. Actually, we did try this approach by aligning those short reads to the regions adjacent to the annotated TSSs instead of using the entire reference genome. However, a large fraction of these reads was still mapped to multiple TSSs rendering impossible further analysis of the short reads. In addition, although the majority of the short reads from σ^{70}-WT and σ^{70}-Δ*greAB* libraries came from annotated transcription start sites, we also observed many σ^{70}-dependent pauses located far away from promoters. These factors hindered tracking the origin of these short reads. At the end, we made a decision to discard them altogether. We highly appreciate this very relevant comment made by the reviewer. We are currently developing a novel NGS approach, which should be able to identify the origin of these short reads.

Re: shorter (12-17nt) RNAs in GreAB-minus libraries (lines 98-99). I do not understand why shorter rather than longer RNAs are observed in the absence of GreA/B. The ms by Marr and Roberts cited here does show shortening of RNAs with GreA/B addition as expected. What is going on here – a competition between sigma70 and GreA/B? Is there anything known about whether GreA/B can actually suppress pausing or is this the finding being made here? This is also talked about in the next section, but there is no indication as to whether this is new or not. The same point is being made in several places throughout, but never decisively. As a side note, it is possible that mapping the aforementioned short reads to TSSs – or proving that they do not map to known TSSs – may help address this.
Response: This result seems not to contradict the known function of GreA/B. First, we did observe a higher ratio of long reads (>= 18-nt) in σ^{70}-Δ*greAB* compared to σ^{70}-WT as shown in Fig. 1c. This difference indicated that the backtracked RNA was not cleaved at the 3' end after deleting greAB. Second, a large fraction of the short (12-17-nt) RNA was observed with high yield in σ^{70}-Δ*greAB* cells due to the strong backtracked G1 pauses that occurred at a short 12-17-nt distance from TSS. The intact 5' end of the RNA was still protected from RNase I in these backtracked complexes. These pauses were more abundant in Δ*greAB* compared to WT cells ultimately enriching the Δ*greAB* dataset with the short reads. As the reviewer mentioned, these RNAs could be shortened by Gre factors in the absence of NTPs as demonstrated by Marr and

Roberts. However, the RNAs cleavage by Gre factors could also reactivate RNAP leading to the rapid extension of the shortened RNAs to the 16-17-nt registers in the presence of NTPs in vivo (Fig. 1c, black column). This competitive RNA extension that is likely to occur in vivo should result in a relatively lower ratio of short (<= 15-nt) and long (>= 18) reads in WT cells. We acknowledged this known observation by adding the following sentence: "Reactivation of backtracked pauses by Gre factors cleavage resulted in RNA extension to the 16-17-nt registers in WT cells." (lines 94-95).

Figure 2C. Enrichment should be also shown normalized against representation of each feature in the genome. Also, what is the significance of showing pauses mapping to coding versus noncoding (presumably 5'-UTR) portions of genes?

Response: We fully understand the reviewer's concern that different share of each feature in the genome should make the pauses to appear more abundant (per 1 kb) in some genomic regions (UTRs vs. ORFs) or in some categories of genes such as coding vs. noncoding genes. Originally, our decision not to show the normalized data in Fig. 2c was based on the following considerations. First, Fig. 2c was introduced to illustrate the differences in the pause site distribution in WT vs. Δ*greAB* cells. Apparently, the normalization step was not essential for this comparison. Secondly, the recent mapping of the transcription start sites in *E. coli* identified a large number of the new sense and antisense promoters with unknown function located in ORFs (Maureen K. T. et al. J Bacteriol. 2015; Laurence E. et al. BMC Genomics. 2016). A substantial faction of the G1 pauses identified in our work localized to these promoters. We wanted to avoid annotating these pauses to ORFs as these promoter may have some functions that was independent of the harboring ORF. Similarly, we did not want to annotate some pauses to 5' vs. 3' UTRs because these feature elements are frequently overlapped in *E. coli* genome. Third, we tried not to use the normalized data, because the high normalized enrichment with a small number of pause sites may artificially inflate biological significance of these pauses on a genome-wide scale. As the reviewer suggested, we normalized the distribution of the pause sites across different genomic regions (as shown below). The Δ*greAB* cells had a larger number of pauses and increased normalized enrichment in untranslated and antisense regions compared to WT cells. We have added these figures to Supplementary Fig. 4 and modified the text accordingly (lines 113-115).

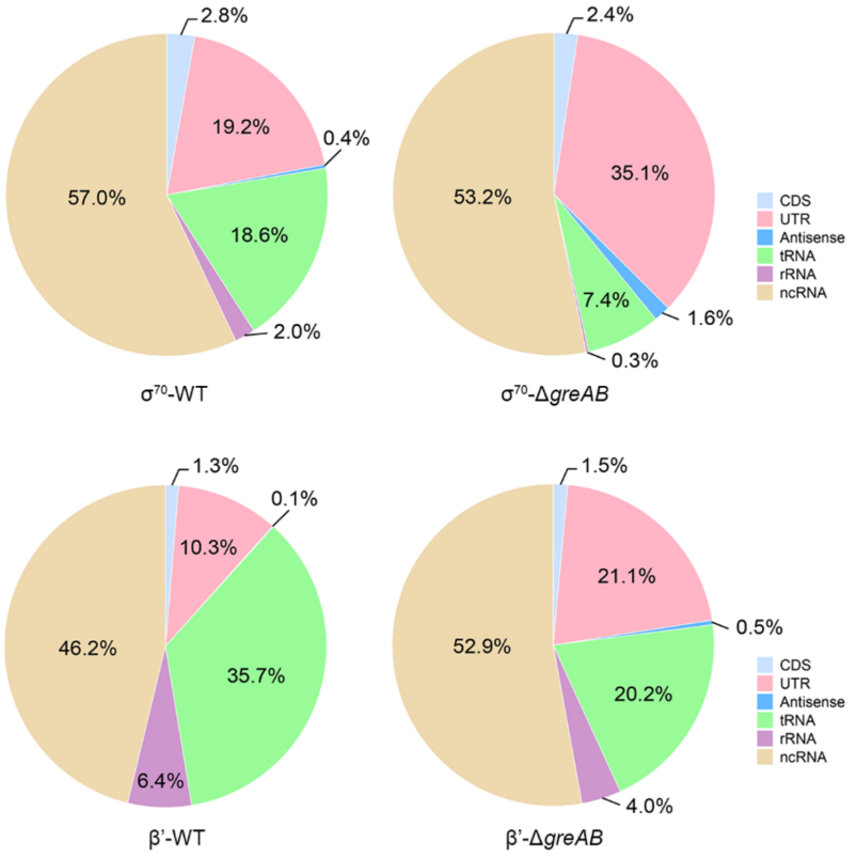

As stated in the text, "a substantial fraction of σ^{70}-dependent pauses was suppressed or released by Gre factors in WT cells. The Δ*greAB* cells had a larger number of pause sites and increased normalized enrichment in untranslated and antisense regions compared to WT cells" (lines 112-115). This analysis suggested the enrichment of σ^{70}-dependent pauses in the UTRs and this was the reason why we analyzed the distances between σ^{70}-dependent pauses and annotated TSS in Fig. 2d.

Based on Figures 2D and 2E, G0 pauses seem to be dominating the wild type sigma70 libraries. This prominence makes them hard to ignore outright. If these are generated by initiation from upstream promoters as suggested in the text (what is meant here by upstream promoter?), why are these transcripts selectively much more sensitive to GreA/B? Why this phenomemon seems to be exclusive to sigma libraries also makes no sense to me.

Response: In our nomenclature, the upstream promoter indicates the promoter located upstream from the nearest annotated TSS, which generates readthrough sense transcription from upstream transcriptional unit. For several G0 pauses that we manually checked in the IGV browser, the upstream promoter appeared to belong to the upstream gene or operon and the G0 pauses were originated from read-through of the upstream terminator. To clarify this point, the text has been modified accordingly (line 126).

We thank the reviewer for raising this important question about the origin of G0 pauses. We were also initially puzzled by the abundance of these pauses in WT cells and their apparent suppression in the Gre-deleted cells. Because the G0 pauses were highly enriched in the σ^{70} libraries, they appeared to belong to σ^{70}-dependent category. However, we noted that these pauses stood out in σ^{70}, but not in β' dataset, which indicated that they comprised only a small fraction of RNAP compared to the regular G1 pauses, diminishing potential impact of the G0 pauses on gene regulation. This is why we decided to put them aside. It's not clear why the G0 pauses were more abundant in σ^{70}-WT. We can only guess that their abundance might directly correlate with the amount of σ^{70} in the cell and the effect of GreA/B on these pauses was indirect. For instance, *E. coli* cells grown in LB were shown to contain 500-1700 molecules of σ^{70} and ~4600 molecules of RNAP core enzyme per cell (Jishage M. J Bacteriol. 1995; Jishage M. J Bacteriol. 1996; Bakshi S. Mol Microbiol. 2012) making the amount of σ^{70} limited compared to core enzyme. When *greAB* were deleted, more σ^{70} became stuck with core at the G1 pauses and less σ^{70} was left for induction of the G0 pauses. The origin of the G0 pauses will be a subject of our investigation in the future.

Comparison of different dinucleotide primers to distinguish contribution of DNA versus RNA is much appreciated. Is there a reason why the Runoff transcript in 4G lane 5 is lower and not higher in intensity than in lane 4 as would be expected if this were due to lower pausing and higher readthrough efficiency?
Response: The dinucleotide used in the reaction of lane 4 (Fig. 4g) shifted the TSS 1 bp upstream from its normal location, which shortened the spacer length between -10R and tssR from 8 to 7 nucleotides. This change may increase strength of the σ^{70} promoters as shown in Fig. 3k (top) resulting in the increased yield of the run-off transcript in lane 4. After normalizing the yield of the runoff transcript, the data of lane 5 showed a lower pausing and higher readthrough efficiency.

Figures 5D and E are extremely hard to understand as presented. First of all, no background DNA reactivity appears to be shown, which would be a serious omission of a control. Secondly, the footprints may be "zoomed in" too much so it is unclear where the bubble begins or ends. Extended figure 15 shows the correct region much more clearly. Is T7A1 a pauseless promoter? If so, it belongs in the main panel to show a contrast with T1 and T3 NTP-dependent reactivity in main 5D.
Response: We agree with the reviewer's suggestion and repeated the $KMnO_4$ footprinting experiments by adding the requested dsDNA control. The new data confirmed our initial observation, and they were included to the updated Fig. 5d, e. The gel autoradiograms are shown below. As the reviewer suggested, the figures showing $KMnO_4$ footprinting results were also zoomed out to clearly show the edges of the transcription bubble.

T7A1 is indeed a pause-less promoter and the corresponding data should be relevant to Fig. 5d as the reviewer mentioned. However, Fig. 5 already contains a large amount of data. To make the main figure easy to read, we decide to keep the $KMnO_4$ footprinting results on T7A1 promoter in the Supplementary Figures.

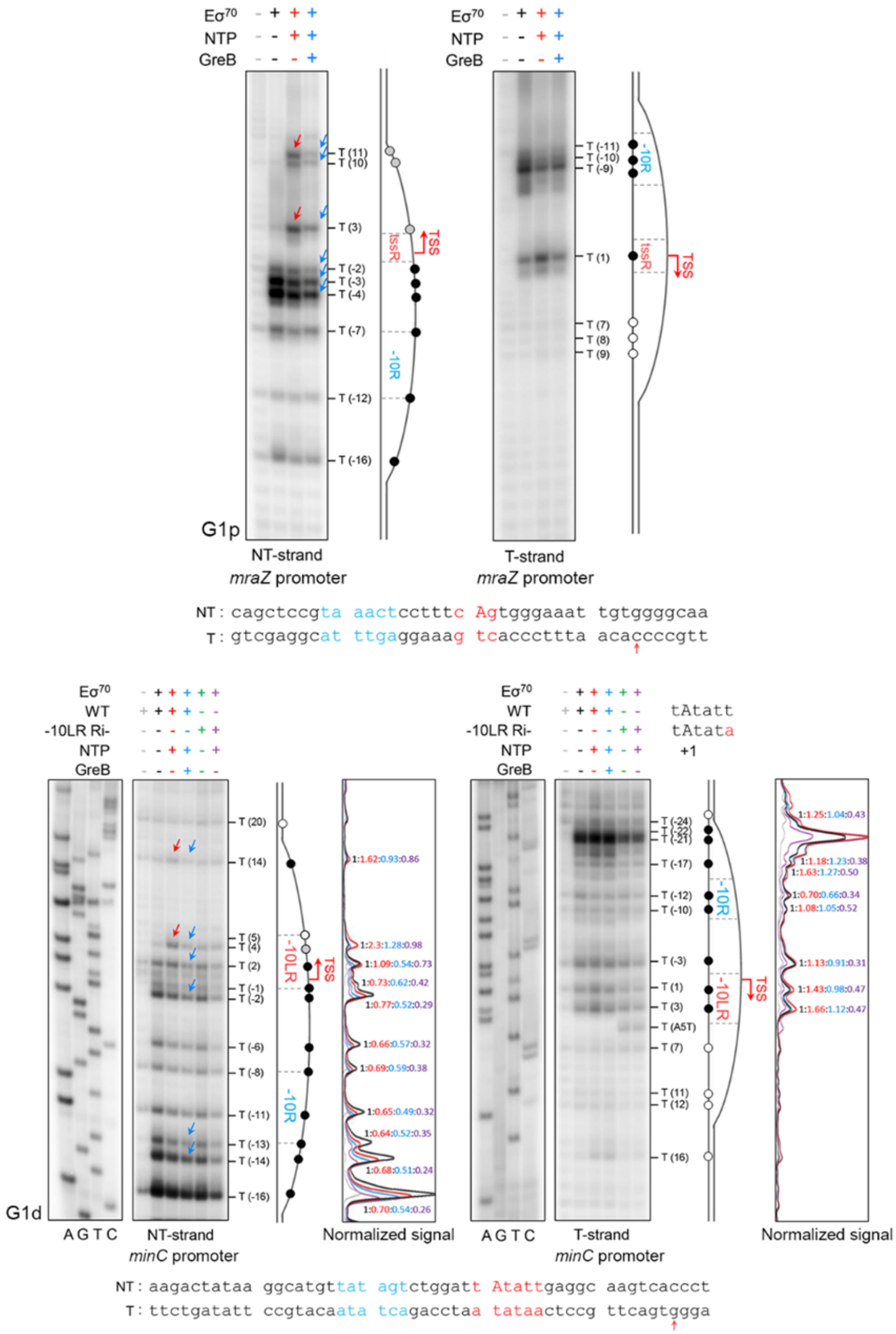

Figure 5E example of paused promoter is particularly hard to understand. Perhaps insert a small arrow to where the difference +/- is? Secondly, the point mutation makes me even more confused. What the mutation may have caused is to create a new promoter there that forms a very nice bubble that is not dependent on NTPs. Given that, I do not know if petering out of green line signal in downstream regions is even real – I do not see it visually on the gel itself.

Response: As the reviewer suggested, we added the red and the blue arrows to depict the modified "T" residues representing the increased and the decreased sensitivity to permanganate of the corresponding DNA bases (the modified figures are shown above). This

modification visualized more clearly the bubble extension to the +14 position (red lane) and the bubble contraction after addition of GreB (blue lane).

We would argue and provide the evidence below that the point mutation reduces the G1 pausing rather than generates a new promoter. This conclusion was also supported by the data shown in Supplementary Fig. 11c, d. Most likely, the impression that the new promoter was generated came from the non-equal loading of the labeled DNA to the gel. We repeated the footprinting experiments and readjusted the volume of the samples to ensure that the same amount of DNA was loaded to the gel. This normalization resulted in the improved visual representation of the footprinting data and revealed that the mutation caused a uniform reduction of the signal from all "T" residues in the transcription bubble. We modified the text (lines 246-248, 251-254) and updated the normalized signal profiles accordingly.

Is there a correlation between the counts of short RNAs at promoters and expression of the same gene? The latter can be probably inferred from beta' datasets in gene bodies if other data are not available. In other words, is pausing repressive or part of a normal transcription cycle?
Response: The G1 pausing was consistently repressive in the absence of GreAB factors. In general, the genes harboring G1 pauses were downregulated in Δ*greAB* cells compared to WT cells (Fig. 6d). This effect was most prominent for the genes coding for the strong G1 pauses, whose corresponding promoter regions had higher counts of short RNET-seq reads (e.g. Fig. 3e, f; top). The RNA-seq further confirmed the lower number of RNA reads immediately downstream from the strong G1 pause sites in Δ*greAB* cells compared to WT cells (e.g. Fig. 3e, f; bottom). Thus, at least some G1 pauses were suppressed or rescued by Gre factors to produce more full-length RNA. According to the reviewer's suggestion, we added this information to the manuscript (lines 154-158).

Figure 1 legend makes speculations about the origin of <16nt RNAs. I wonder if this material should be in Discussion rather than figure legends, but this is of course up to the authors. In followup future experiments these may be possible to tell apart by ligating RNA pools +/- alkaline phosphatase pre-treatment.
Response: Thanks for the reviewer's suggestion. However, our manuscript primarily focuses on the σ^{70}-dependent pausing, but not on a source of the short RNA reads per se. Thus, we find more reasonable to keep this material in the figure legend rather than in Discussion. In fact, our in vitro footprinting data showed that non-backtracked RNAP holoenzyme typically protects 16-17-nt segment of the nascent RNA from RNase I digestion (Supplementary Fig. 2). Our RNET-seq data strongly indicated that a large fraction of the short <16-nt RNA species particularly enriched in σ^{70}-Δ*greAB* dataset derived from strong backtracked G1-like pauses where the native 5' end of the RNA is not yet accessible to RNase I. These results established a correlation of the in vitro footprinting data, RNET-seq data and the origin of these <16-nt RNAs. We voluntarily placed this speculation to Fig. 1 legend not to distract the readers from our major findings. As the reviewer mentioned, we are currently trying to make the libraries derived from the short 5' triphosphate-carrying RNAs.

Reviewer #1 (Remarks to the Author):

Whilst it would have been nice to have a more in-depth RNAseq analysis investigating the Gre controlled expression of transcriptional regulators, I appreciate the authors comments on why that's not feasible.

All of my other comments have been taken on board and addressed, which I thank the authors for.

Reviewer #2 (Remarks to the Author):

The authors appear to have answered adequately the comments and suggestions of the three reviewers, and have provided a few new experiments as well. The paper will be a worthy development of the subject, and no doubt will stimulated further work on the nature and function of sigma-dependent pausing. It is now appropriately prepared for publication.

Reviewer #3 (Remarks to the Author):

In a revision, Sun et al largely addressed my comments. While some of the issues raised do not have clearcut answers, they are, in my opinion, should be left to argue by the scientific community after dissemination. The data are solid. I especially appreciate the authors providing updated permanganate footprints that show the effects much more clearly and leave little room for ambiguity.
A couple of points that are mainly editorial:
Line 21 – the first sentence of abstract – check for grammar.
Line 81 – in addition to referring to Fig. 1b, add the citation (Ref7) that was mentioned in a rebuttal.
Line 94-95. The newly inserted sentence is appreciated, but should be toned down (for example, by using wording such as “may have” or “we suggest that”).
Line 104. A word might be missing in “recovered by affinity” – should be affinity pull-down or immunoprecipitation, or something like that.
Line 247 – a word is missing in the newly inserted “to that was”
Line 247 – delete “and”
Line 254 Should be “inactive” or “inactivated”
Line 266. Insert “may have” before “dissociated” to tone the sentence down
Line 298. The word “process” appears to be omitted. Also check grammar in 301-302.

Reviewer #1 (Remarks to the Author):

Whilst it would have been nice to have a more in-depth RNAseq analysis investigating the Gre controlled expression of transcriptional regulators, I appreciate the authors comments on why that's not feasible.
Response: We highly appreciate the reviewer's comment. First, the expression of several transcriptional regulators controlled by Gre factors was included to Fig. 6d. Second, although the genes coding for transcription regulators controlled by G1 pausing were significantly enriched in the Gre-dependent pool, their fraction was relatively low (8.4%). This is why we made a decision not to perform their separate analysis.

All of my other comments have been taken on board and addressed, which I thank the authors for.
Response: We are thankful to the reviewer for the helpful and positive comments.

Reviewer #2 (Remarks to the Author):

The authors appear to have answered adequately the comments and suggestions of the three reviewers, and have provided a few new experiments as well. The paper will be a worthy development of the subject, and no doubt will stimulated further work on the nature and function of sigma-dependent pausing. It is now appropriately prepared for publication.
Response: We appreciate the reviewer's insights and valuable comment.

Reviewer #3 (Remarks to the Author):

In a revision, Sun et al largely addressed my comments. While some of the issues raised do not have clearcut answers, they are, in my opinion, should be left to argue by the scientific community after dissemination. The data are solid. I especially appreciate the authors providing updated permanganate footprints that show the effects much more clearly and leave little room for ambiguity.
Response: We appreciate the reviewer's helpful and valuable comments on our work.

A couple of points that are mainly editorial:
Line 21 – the first sentence of abstract – check for grammar.
Response: This sentence has been modified (lines 21-22).

Line 81 – in addition to referring to Fig. 1b, add the citation (Ref7) that was mentioned in a rebuttal.
Response: The citation has been added (line 82).

Line 94-95. The newly inserted sentence is appreciated, but should be toned down (for example, by using wording such as "may have" or "we suggest that").
Response: The statement was toned down as the reviewer suggested (lines 95-96).

Line 104. A word might be missing in “recovered by affinity” – should be affinity pull-down or immunoprecipitation, or something like that.
Response: We thank the reviewer for thorough reading of the manuscript. The word “pull-down” was added to the sentence (line 105).

Line 247 – a word is missing in the newly inserted “to that was”
Response: The sentence has been modified (line 248).

Line 247 – delete “and”
Response: It has been deleted as suggested.

Line 254 Should be “inactive” or “inactivated”
Response: It has been modified as suggested (line 255).

Line 266. Insert “may have” before “dissociated” to tone the sentence down
Response: We have modified the sentence accordingly (line 267).

Line 298. The word “process” appears to be omitted. Also check grammar in 301-302.
Response: The word “process” was added to the sentence (line 301) and the grammar was also fixed (line 303-305).
